# Petrosal Anatomy of the Paleocene Eutherian Mammal *Deltatherium fundaminis* (Cope, 1881)

**DOI:** 10.1007/s10914-021-09568-3

**Published:** 2021-08-31

**Authors:** Sarah L. Shelley, Ornella C. Bertrand, Stephen L. Brusatte, Thomas E. Williamson

**Affiliations:** 1 School of GeoSciences, University of Edinburgh, Edinburgh, United Kingdom; 2 Carnegie Museum of Natural History, Pittsburgh, Pennsylvania United States of America; 3 New Mexico Museum of Natural History and Science, Albuquerque, New Mexico United States of America

**Keywords:** Paleogene, *Deltatherium*, Auditory region, Condylarthra, Basicranium

## Abstract

We describe the tympanic anatomy of the petrosal of *Deltatherium fundaminis,* an enigmatic Paleocene mammal based on cranial specimens recovered from New Mexico, U.S.A. Although the ear region of *Deltatherium* has previously been described, there has not been a comprehensive, well-illustrated contribution using current anatomical terminology. The dental and cranial anatomy of *Deltatherium* is a chimera, with morphological similarities to both ‘condylarth’ and ‘cimolestan’ taxa. As such, the phylogenetic relationships of this taxon have remained elusive since its discovery, and it has variably been associated with Arctocyonidae, Pantodonta and Tillodontia. The petrosal of *Deltatherium* is anteriorly bordered by an open space comprising a contiguous carotid opening and pyriform fenestra. The promontorium features both a small rostral tympanic process and small epitympanic wing but lacks well-marked sulci. A large ventral facing external aperture of the canaliculus cochleae is present and bordered posteriorly by a well-developed caudal tympanic process. The hiatus Fallopii opens on the ventral surface of the petrosal. The tegmen tympani is mediolaterally broad and anteriorly expanded, and its anterior margin is perforated by a foramen for the ramus superior of the stapedial artery. The tympanohyal is small but approximates the caudal tympanic process to nearly enclose the stylomastoid notch. The mastoid is widely exposed on the basicranium and bears an enlarged mastoid process, separate from the paraoccipital process. These new observations provide novel anatomical data corroborating previous hypotheses regarding the plesiomorphic eutherian condition but also reveal subtle differences among Paleocene eutherians that have the potential to help inform the phylogeny of *Deltatherium.*

**Supplementary Information:**

The online version contains supplementary material available at 10.1007/s10914-021-09568-3.

## Introduction

*Deltatherium,* meaning ‘triangular-beast’ in reference to its tritubercular teeth, is a eutherian mammal known from Paleocene-aged deposits in the San Juan Basin of New Mexico, U.S.A (Cope 1881a; Matthew 1937; Williamson 1996). At first glance, the fossils of *Deltatherium* allude to a rather innocuous opossum-sized animal with dental and cranial adaptations indicative of an omnivorous to carnivorous diet and a disproportionately large and robust head compared to its scantily known postcrania (Cope 1881a; Matthew 1937). However, closer consideration of *Deltatherium* reveals it to be among the most enigmatic Paleocene mammals, a chimera of morphologies similar to both ‘condylarth’ and ‘cimolestan’ taxa. As such, its phylogenetic relationships have remained elusive since its discovery. Determining the phylogenetic position of *Deltatherium* is a key step in resolving broader issues of Paleocene mammal phylogeny and requires detailed anatomical descriptions to inform character data.

The specific objective of this study is to describe the tympanic petrosal anatomy of *Deltatherium fundaminis*. Matthew (1937) briefly described the auditory region of *Deltatherium* in his seminal posthumous monograph on the Paleocene faunas of the San Juan Basin. Here, we update and expand upon Matthew’s description and include detailed comparisons to other Paleogene mammals. The petrosal bone is a rich source of comparative anatomical data and continues to be a useful source of phylogenetic information. Nevertheless, since it is not the goal of this paper to resolve the phylogenetic position of *Deltatherium*, we do not lend credence to any phylogenetic hypotheses at present and refrain from making any statements regarding that matter. We will, however, describe and discuss the comparative anatomy of *Deltatherium* within a phylogenetic context, and we intend to use information collected from this study in future work evaluating the phylogenetic relationships of Paleocene mammals.

### Historical Background

The taxonomy and systematics of *Deltatherium* have a long history of study; however, it is useful to detail this information to understand why the phylogenetic affinities of this taxon have remained so elusive. *Deltatherium* was first described in 1881 by E. D. Cope based on the upper dentition (Cope 1881a). The upper dentition indicated a more omnivorous-carnivorous diet, and Cope considered *Deltatherium* a member of Leptictidae within Creodonta rather than a member of ‘Condylarthra’, which was established based on taxa with more bunodont dentitions and tendencies towards a more omnivorous-herbivorous diet (Cope 1881a). In the same year, Cope described the lower dentition of *Deltatherium* based on a partial dentary preserving the three lower molars; he named it *Lipodectes penetrans* and considered it to be a mesonychid creodont (Cope 1881b). With the discovery of new material, Cope recognised the association between the upper and lower dentition of *Deltatherium* and reasserted a leptictid classification within Creodonta (Cope 1884).

In 1887, M. Schlosser revised Creodonta, moving *Deltatherium* from Leptictidae to Proviverridae which also included *Triisodon* and *Didelphodus* (Schlosser 1887). Cope acknowledged Schlosser’s referral of *Deltatherium* to Proviverridae and also referred *Mioclaenus, Onychodectes*, *Triisodon* and *Chriacus* to the group (Cope 1888). W. B. Scott retained Cope’s concept of Proviverridae except for moving *Chriacus* to Oxyclaenidae (Scott 1892). H. F. Osborn and C. Earle followed suit and retained a Proviverridae classification (Osborn and Earle 1895)*.* W. D. Matthew was indirectly skeptical that *Deltatherium* pertained to Proviverridae (Matthew 1897), and in 1899, he shifted *Deltatherium* from Proviverridae to Oxyclaenidae within Creodonta and alluded to a close affinity with *Chriacus* (Matthew 1899). In 1937, Matthew reviewed the taxonomic history of *Deltatherium* in detail and described the specimen studied here, which was found during the 1913 expeditions to the San Juan Basin (Matthew 1937). Matthew transferred *Deltatherium* to the subfamily Chriacinae within Arctocyonidae, which was still considered a member of Creodonta at the time.

Following a mid-century hiatus, C. L. Gazin noted a number of similarities between *Deltatherium* and members of Tillodontia as well as some pantodonts (Gazin 1953). Tillodonts are medium to large-sized eutherian mammals known from North America and Asia distinguished by their rodent-like second upper incisors and other features more reminiscent of carnivores or ungulates (Gazin 1953; Lucas and Schoch 1998; Rose 2006). Pantodonts are mostly medium to large-sized herbivorous mammals known from North and South America and Asia and were among the first eutherian mammals to attain truly large body sizes following the end-Cretaceous mass extinction (Simons 1960; Lucas 1998; Rose 2006). E. L. Simons (1960) also alluded to some shared, potentially plesiomorphic, features of the dentition and basicranium of *Deltatherium* and members of Pantodonta. Regarding the auditory region, Simons noted that both *Deltatherium* and *Titanoides* shared a broad, expanded region on the anterointernal surface of the petrosal beyond the region of the promontorium, which houses the cochlea (Simons 1960). It is likely that Simons was referring to the rostral tympanic process and medial shelf.

L. Van Valen (1978) went a step further and hypothesised a pantodont affinity for *Deltatherium.* He speculated on a close relationship with the arctocyonid *Oxyprimus* (a member of the Oxyclaeninae) and illustrated *Deltatherium* giving rise to Pantodonta (Van Valen 1978). It is worth noting that Arctocyonidae had been transferred from Creodonta to ‘Condylarthra’, a position that is still broadly upheld, prior to Van Valen hypothesizing on the origins of pantodonts (Simpson 1936; Gazin 1941; Van Valen 1966, 1969). Van Valen did not provide any explanation for his hypothesis but implied some dental characteristics shared by *Deltatherium* and early Torrejonian pantodonts such as *Pantolambda.* Cifelli (1983) questioned an arctocyonid/pantodont affinity for *Deltatherium*, instead proposing a broadly defined ‘insectivore’ affinity for the taxon, possibly alluding to a cimolestan ancestry*.* McKenna (1975) had previously included *Deltatherium* within Pantodonta, which he classified within Cimolesta, but did not provide any rationale for his assertion. Szalay (1977) disputed McKenna’s cimolestan classification of Pantodonta and provided discussion in support of Van Valen’s arctocyonid-*Deltatherium-*pantodont hypothesis based on dental and postcranial observations. Shortly thereafter, Chow et al. (1977) proposed a close relationship between pantodonts and tillodonts within Cimolesta. Van Valen (1988) subsequently reasserted an arctocyonid affinity for *Deltatherium* and erected the subfamily Deltatheriinae within Arctocyonidae, which he considered closely related to Pantodonta, presumably based on dental similarities, although this was not explicitly stated.

Muizon and Marshall (1992) dismissed a close relationship between *Deltatherium* and pantodonts when comparing *Deltatherium* to *Alcidedorbignya*, an early Paleocene pantodont from Bolivia. Lucas (1993) noted a close resemblance between *Deltatherium* and early tillodonts and proposed a sister taxon relationship within Cimolesta and potentially a close affinity with *Didelphodus* (Lucas 1993; Lucas and Schoch 1998; Lucas and Kondrashov 2004). Zack (2010) conducted a comprehensive higher-level phylogenetic analysis of Paleogene eutherians and found *Deltatherium* to be the sister taxon to a clade including numerous arctocyonids (*Chriacus*, *Thryptacodon*, *Claeonodon*, *Mentoclaenodon*, and *Anacodon*) and the extinct pangolin *Patriomanis* (constrained analysis), and he commented that a close relationship with pantodonts and tillodonts was unlikely. Other recent studies have continued to refer to *Deltatherium* when discussing arctocyonids, pantodonts, or tillodonts (e.g. Rose 2006; Muizon et al. 2015) but have refrained from inferring the phylogenetic affinities of *Deltatherium*.

At present, two species of *Deltatherium* are currently recognised: *Deltatherium fundaminis* from San Juan Basin Mammal biozone Tj4-Tj5 and *Deltatherium dandreae* from Tj2-Tj3 (Williamson 1996; Lucas and Kondrashov 2004). Van Valen (1978) named a third species of *Deltatherium*, *Deltatherium durini*, based on a molariform tooth from the Crazy Mountains Basin locality in Montana, but this was later found to be a viverravid deciduous premolar (Zack 2012). Currently, there is no clear consensus regarding the phylogenetic position of *Deltatherium*, and an affinity with Arctocyonidae, Pantodonta, and/or Tillodontia remain entirely plausible – a ‘triangular beast’ indeed. Collectively, these studies highlight the need for detailed anatomical observations of key taxa such as *Deltatherium* to provide character data for inclusion in phylogenetic analyses*.*

## Materials and Methods

**Fig. 1:**
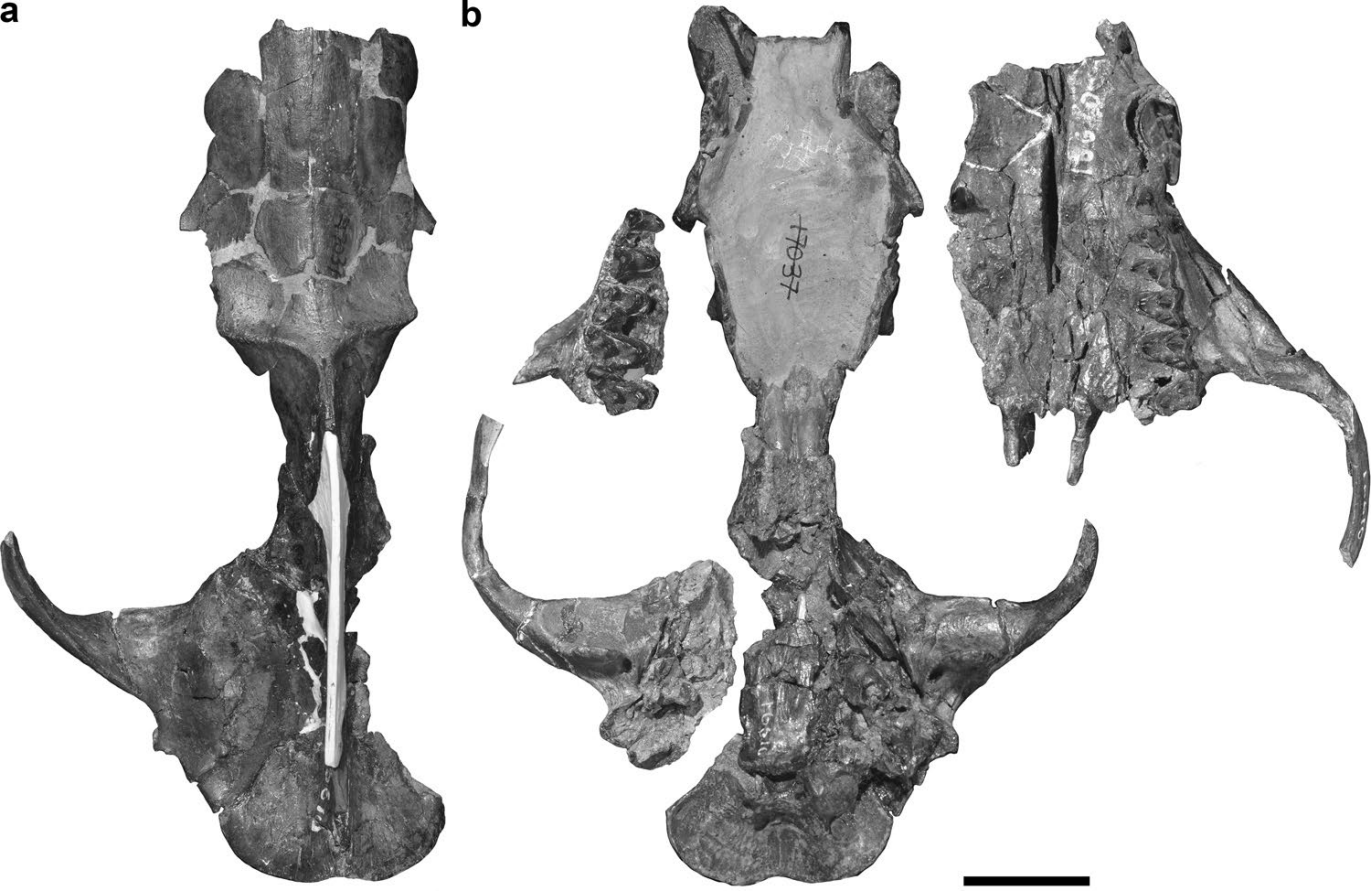
Photographs of the cranium of *Deltatherium fundaminis* (AMNH 16610) in **a**, dorsal view; **b**, ventral view. Scale bar = 20 mm

**Fig. 2:**
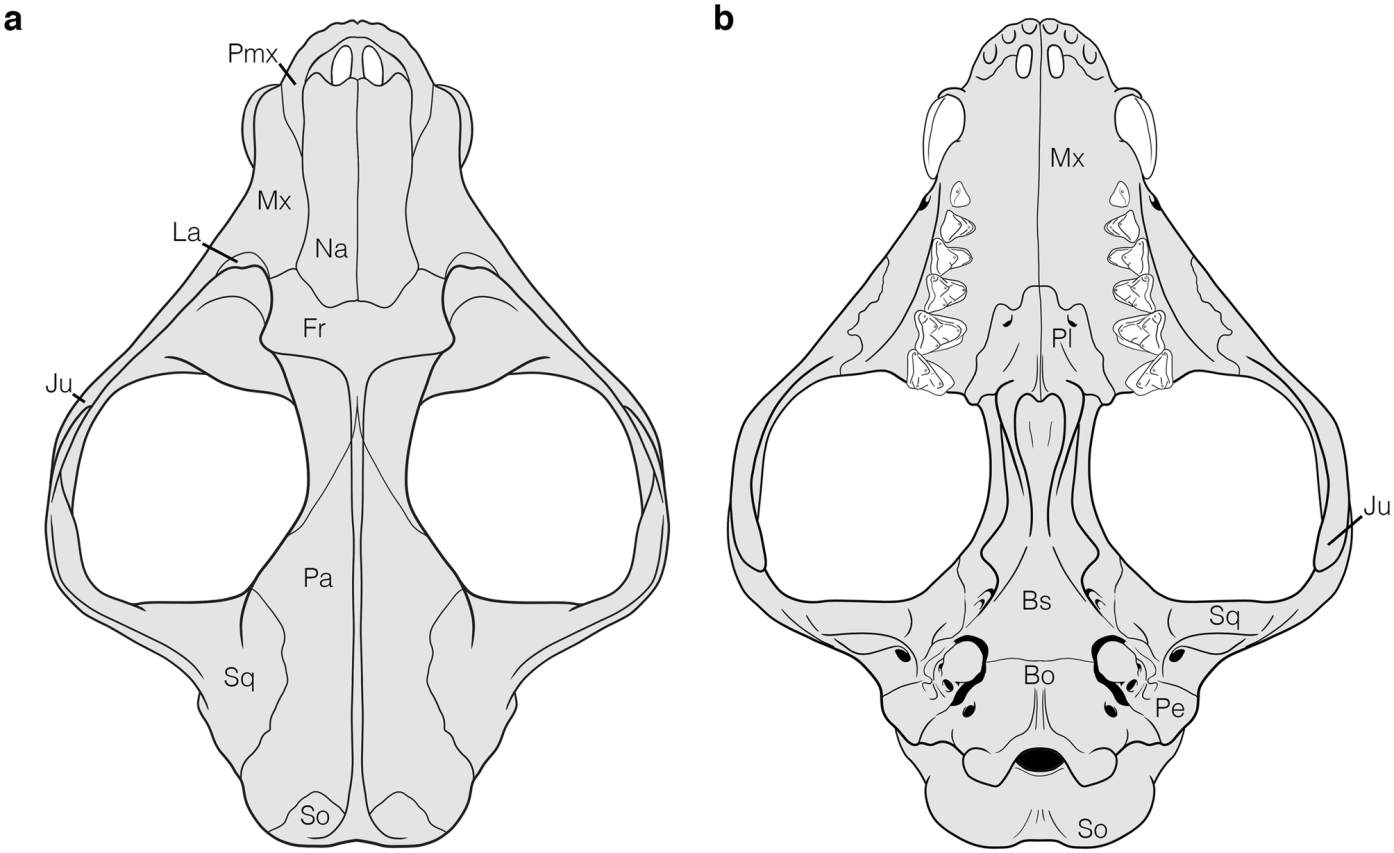
Annotated line drawings of the reconstructed cranium of *Deltatherium fundaminis* (AMNH 16610) in **a**, dorsal view; **b**, ventral view. Reconstruction based on specimen photographs in Fig. 1 with reference to the figures in Matthew (1937). Abbreviations: Bo, basioccipital; Bs, basisphenoid; Fr, frontal; Ju, jugal; La, lacrimal; Mx, maxilla; Na, nasal; Pa, parietal; Pe, petrosal; Pl, palatine; Pmx, premaxilla; So, supraoccipital; Sq, squamosal

**Fig. 3:**
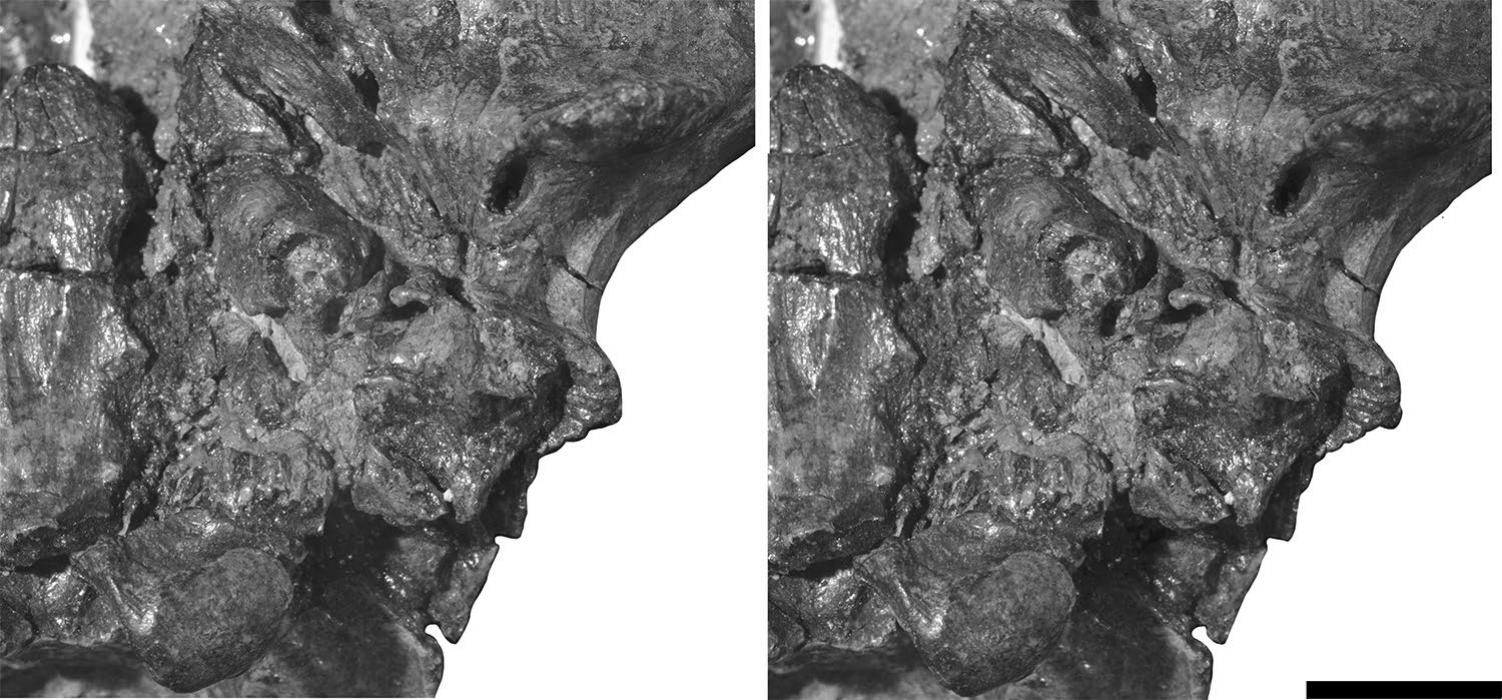
Stereophotographs of left auditory region of *Deltatherium fundaminis* (AMNH 16610). Scale bar = 10 mm

**Fig. 4:**
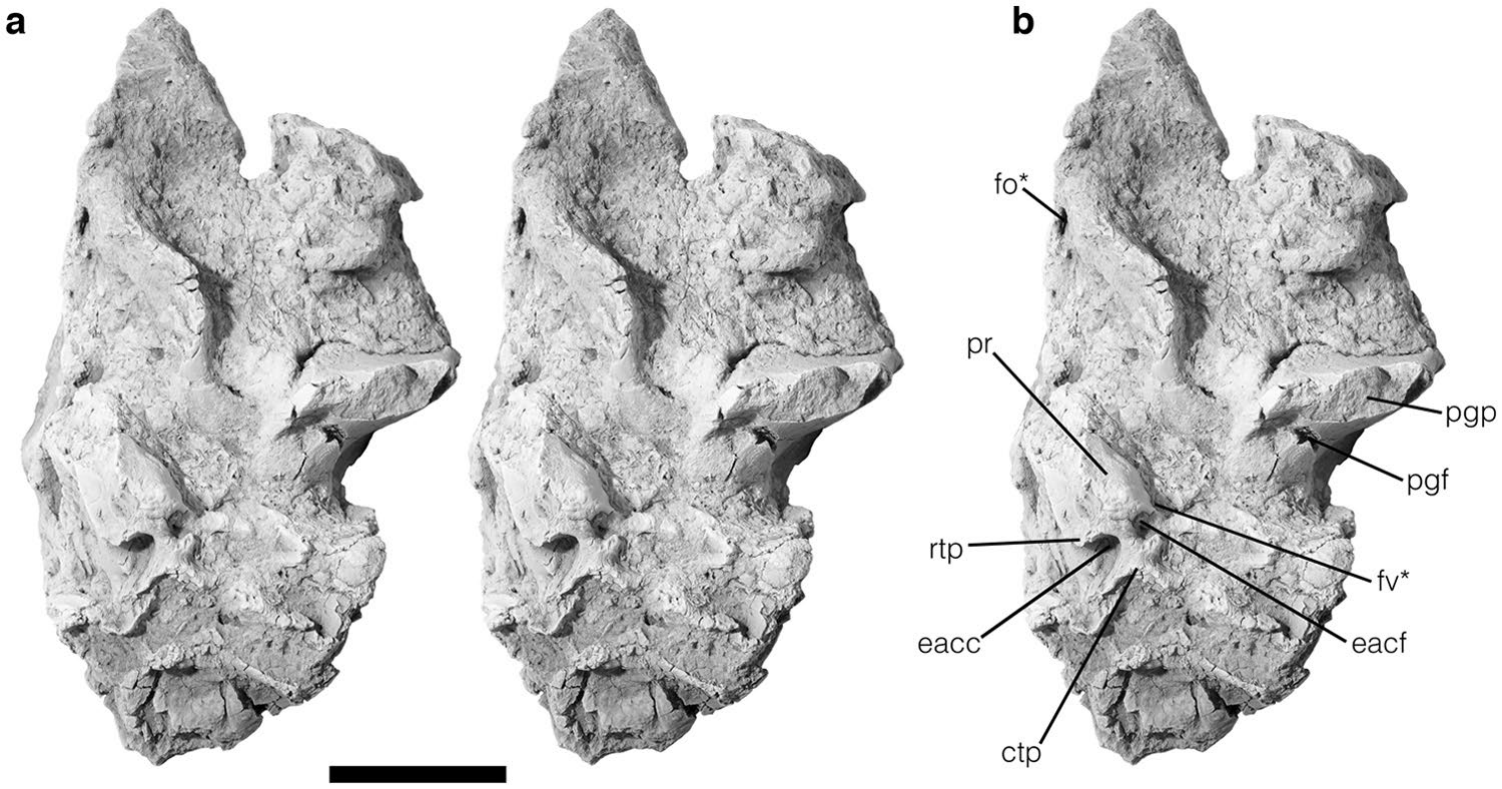
The left auditory region of *Deltatherium fundaminis* (NMMNH P-54104). **a**, stereophotographs of the specimen which has been coated in magnesium oxide; **b**, annotated photograph. Abbreviations: ctp, caudal tympanic process; eacc, external aperture of the canaliculus cochleae; eacf, external aperture of the cochlear fossula; fo, foramen ovale; fv, fenestra vestibuli; pgf, postglenoid foramen; pgp, postglenoid process; pr, promontorium; rtp, rostral tympanic process; * denotes damage. Scale bar = 10 mm

This study describes the auditory region of *Deltatherium fundaminis* based on two specimens. The first is a near complete cranium, AMNH 16610, from Torrejonian strata of the Nacimiento Formation in the San Juan Basin, New Mexico (Matthew 1937) (Figs. 1, 2 and 3; Online Resource 1, Fig. S1). The specimen was collected on September 1^st^ by George Olsen from the East Flank of Torreon Wash during the 1913 expeditions led by Walter Granger (Lucas and Estep 1997). High resolution magnetostratigraphy of the Nacimiento Formation gives an age of ~62.6 million years old for the specimen correlating to the Torrejonian (To2) North American Land Mammal ‘Age’ (NALMA) equivalent to San Juan Basin Mammal Biozone, Tj4 (Williamson 1996; Leslie et al. 2018). The second specimen, NMMNH P-54104 (Fig. 4), is fragmentary skull which preserves a partial upper and lower dentition and a piece of the cranium preserving the petrosal with a part of the squamosal. The specimen was recovered from the Bab’s Basin locality (L-06419) (~62.7 Ma, To2/Tj4) in the San Juan Basin (Leslie et al. 2018).

Throughout the descriptive text, comparisons are made to a selection of eutherian taxa. These include the Cretaceous eutherians *Zalambdalestes* and *Asioryctes*, the Cretaceous(?)-Paleocene eutherian *Protungulatum* (Online Resource 1, Fig. S2)*,* the Paleocene arctocyonids *Chriacus* (Online Resource 1, Fig. S3) and *Arctocyon* (Online Resource 1, Fig. S4), the Paleocene pantodonts *Alcidedorbignya* and *Pantolambda* (Online Resource 1, Fig. S5)*,* and the late Paleocene-Eocene tillodonts *Esthonyx* and *Trogosus* (Online Resource 1, Fig. S6). In these comparisons, *Zalambdalestes lechei* serves as an exemplar of the primitive eutherian condition. *Zalambdalestes* is a zalambdalestid eutherian mammal known from the Upper Cretaceous Djadokhta Formation (lower Campanian) of the Gobi Desert, Mongolia (Kielan-Jaworowska 1984). Comparisons are made to PSS-MAE 130, a near complete cranium including the left and right auditory regions that is described and illustrated by Wible et al. (2004). We also make comparisons to *Asioryctes nemegtensis*, an asioryctid eutherian mammal known from the Upper Cretaceous Baruungoyot Formation (upper Campanian) of the Gobi Desert, Mongolia based on a nearly complete skull, ZPAL MgM-I/98. The specimen was described by Kielan-Jaworowska (1981) and more recently included in the study of Wible et al. (2004).

*Protungulatum* is a eutherian mammal known from Cretaceous-Paleogene deposits of North America (Archibald et al. 2011) and has been considered a basal member of Arctocyonidae (Sloan and Van Valen 1965; Van Valen 1978). Other studies have excluded *Protungulatum* from Arctocyonidae (Prothero et al. 1988) and considered it as the oldest undisputed species within crown Placentalia (O’Leary et al. 2013) or, in some cases, found it to be a non-placental stem eutherian (Wible et al. 2007, 2009; Archibald et al. 2011; Halliday et al. 2017). Regardless of its phylogenetic affinities, *Protungulatum* is a basal ‘archaic ungulate’ (O’Leary et al. 2013). Comparisons are made to AMNH 118359 (Online Resource 1, Fig. S2), an isolated left petrosal recovered from the Bug Creek Anthills locality of northeastern Montana (Sloan and Van Valen 1965) that has been referred to *Protungulatum* sp. (MacIntyre 1972; Wible et al. 2007, 2009)*.* It was more recently illustrated and described by O’Leary (2010) and Orliac and O’Leary (2016)*.*

We make comparisons to two putative arctocyonids. *Chriacus pelvidens* was initially placed by Cope in the same genus as *Deltatherium* under the name *Lipodectes pelvidens* (Cope 1881c), and the taxonomic and systematic histories of the two taxa are intertwined, although *Chriacus* is now widely considered a member of Arctocyonidae (e.g. Archibald 1998), whereas the affinities of *Deltatherium* remain unresolved. We make comparisons with the isolated petrosals of *Chriacus pelvidens* illustrated in Bertrand et al. (2020) (NMMNH P-62258; Online Resource 1, Fig. S3). The petrosals were found in association with cranial, dental, and postcranial remains also recovered from the East Flank of Torreon Wash, likely in close proximity to the location where the *Deltatherium* cranium was found and from the same stratigraphic horizon (~62.6 Ma; Leslie et al. 2018). A partial left dP5 was recovered, indicating the specimen represents a juvenile individual. *Arctocyon primaevus* is a relatively large-bodied arctocyonid known from the late Paleocene of Europe. This species was first described by Blainville (1841), and the auditory region was subsequently described by Russell (1964). We include *Arctocyon* as an exemplar of a larger-bodied arctocyonid with dental and cranial adaptations towards carnivory. Comparisons are made to MNHN BR L9, CRL 957 (Online Resource 1, Fig. S4), and UCMP 61454, near complete crania preserving the left and right auditory regions.

With respect to pantodonts, we make detailed comparisons with *Alcidedorbignya inopinata* and *Pantolambda bathmodon. Alcidedorbignya* is a small-bodied pantodont from the Tiupampan South American Land Mammal Age of the Santa Lucia Formation at Tiupampa, Bolivia, approximately equivalent to Torrejonian 1 (To1) of North America (Muizon and Marshall 1992; Muizon et al. 2015). It is known from exceptional fossils including several crania that preserve the auditory region. For our comparisons, we refer to MHNC 8372 and MHNC 8399, both of which were figured and described by Muizon et al. (2015). *Pantolambda* is a medium-sized pantodont also known from the Torrejonian of the San Juan Basin that is nearly contemporaneous with *Deltatherium*. Our comparisons are based on AMNH 16663 (Online Resource 1, Fig. S5), a nearly complete skeleton that includes the cranium with both petrosals. The specimen was described by Matthew (1937), and the auditory region was redescribed by Muizon et al. (2015) following further preparation of the specimen.

Finally, we make comparisons with the two tillodonts, *Esthonyx ancylion* and *Trogosus castoridens. Esthonyx* is a medium-sized tillodont known from the Clark’s Fork Basin, Wyoming that dates to the late Paleocene (Clarkforkian) (Gingerich and Gunnell 1979)*.* The generic assignment of *Esthonyx ancylion* is debated. Gingerich (1989) assigned all Clarkforkian species of *Esthonyx* with a double-rooted p2 and an unfused mandibular symphysis to a new genus, *Azygonyx*, resulting in the new combination *Azygonyx ancylion.* However, Lucas and Schoch (1998) disputed generic distinction based on the characters provided in Gingerich (1989) and recognized *Esthonyx ancylion* as the valid name. We retain the name *Esthonyx ancylion* to be congruent with previous literature referenced in this study, namely Gingerich and Gunnel (1979). Our comparisons are based on UM 68511, two mandibles and a cranium preserving the left and right auditory regions (albeit not particularly well). *Trogosus* is a more recent tillodont known from the Delmar Formation in San Diego County, California, dating to the early Eocene (Bridgerian). We make comparisons with *Trogosus hillsi* (USNM 17157, Online Resource 1, Fig. S6) and *Trogosus castoridens* (SDSNH 40819) (Miyata and Deméré 2016). *Trogosus* is slightly younger geologically than most other taxa to which we make comparisons but preserves the auditory region in greater detail than *Esthonyx.*

Measurements were made using digital callipers, in millimetres, to the nearest two decimal places. Digital measurements were taken with ImageJ (Schneider et al. 2012). The cranium of *Deltatherium* (AMNH 16610) was scanned prior to the COVID-19 pandemic, but the presence of x-ray opaque minerals precluded digital reconstruction of its endocranial anatomy from the data available at the time of study. We hope to remedy this in the future with additional CT work.

The anatomical terminology follows Van der Klaauw (1931), De Beer (1937), McDowell (1958), MacPhee (1981), and the many works of John Wible, whose descriptive studies of basicranial anatomy set a standard of excellence and in whose honor we write this paper (Wible 2003, 2008, 2010, 2011, 2012; Wible and Shelley 2020). Usage of English equivalents of the Nomina Anatomica Veterinaria (International Committee Veterinary Gross Anatomical Nomenclature 2017) is preferred when appropriate following Wible (2003, 2008). For certain anatomical systems, particularly vasculature, the Nomina Anatomica Veterinaria is not adequate because its comparative base is not sufficiently broad, and we employ the terminology of Wible (1984, 1987).

Institutional Abbreviations:

AMNH, American Museum of Natural History, New York, New York, U.S.A.

MHNC, Museo de Historia Natural “Alcide d’Orbigny”, Cochabamba, Bolivia.

MNHN, Muséum national d’Histoire naturelle, Paris, Collection de Paléontologie, Paris, France.

NMMNH, New Mexico Museum of Natural History and Science, Albuquerque, New Mexico, U.S.A.

PSS-MAE, Paleontological and Stratigraphy Section (Geological Institute), Mongolian Academy of Sciences, Ulaan Baatar, Mongolia – American Museum of Natural History Expeditions.

SDSNH, San Diego Natural History Museum, San Diego, California, U.S.A.

UCMP, University of California Museum of Paleontology, Berkeley, California, U.S.A.

USNM, Smithsonian National Museum of Natural History, Washington DC, U.S.A.

UM, University of Michigan Museum of Paleontology, Ann Arbor, Michigan, U.S.A.

ZPAL, MgM Institute of Paleobiology, Polish Academy of Sciences, Warsaw, Poland.

## Description

### Specimen Overview

The cranium of *Deltatherium fundaminis*, AMNH 16610, is broken into four pieces (Figs. 1 and 2): (1) the principal piece, including the dorsal half of the rostrum, the mesocranium, the left auditory region with the posterior root of the left zygomatic arch, and the occiput; (2) the ventral part of the palate, including the upper left postcanine dentition and the anterior portion of the zygomatic arch; (3) a small piece preserving the upper right postcanine dentition and part of the right maxilla; (4) the right auditory region and the posterior portion of the right zygomatic arch.

The left auditory region is better preserved than the right; the left petrosal is in situ, but the basicranial bones surrounding it are displaced. The basisphenoid is fractured, and the central portion that contacts the basioccipital is shifted medially. A part of the alisphenoid is preserved but is not in contact with the basisphenoid. The basioccipital comprises part of the basicranial exposure of the occipital with the exoccipital and is not well preserved; however, the margins and sutures are discernible. The squamosal has shifted slightly laterally relative to the petrosal. Consequently, the sutures among the basisphenoid, alisphenoid, and squamosal are no longer in life position, though they can be reconstructed since the specimen is well enough preserved. The ventral surface of the right petrosal is damaged, but the squamosal-petrosal suture is well delimited, and the margins of the petrosal are discernible.

**Fig. 5:**
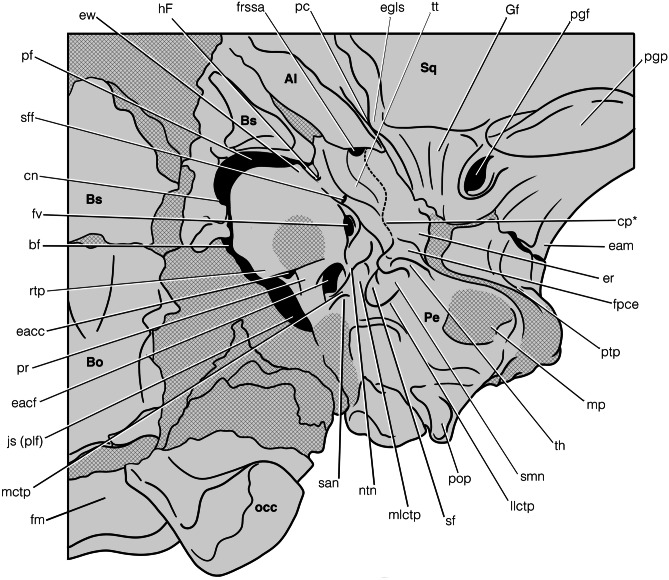
Annotated line drawing of the left auditory region of *Deltatherium fundaminis* (AMNH 16610). Abbreviations: Al, alisphenoid; bf, basicapsular fissure; Bo, basioccipital, Bs, basisphenoid; cn, carotid notch; cp, crista parotica; eacc, external aperture of the canaliculus cochleae; eacf, external aperture of the cochlear fossula; eam, external acoustic meatus; egls, entoglenoid process of the squamosal; er, epitympanic recess; ew, epitympanic wing; fpce, facet for the posterior crus of the ectotympanic; fm, foramen magnum; frssa, foramen for the superior ramus of the stapedial artery; fv, fossula for the fenestra vestibuli; Gf, Glaserian fissure; hF, hiatus Fallopii; js (plf), jugular sulcus (posterior lacerate foramen); llctp, lateral part of the lateral caudal tympanic process; mctp, medial caudal tympanic process; mlctp, medial part of the lateral caudal tympanic process; mp, mastoid process; ntn, notch for the tympanic nerve (branch of the glossopharyngeal nerve, CN IX); occ, occipital condyle; pc, preotic crest; Pe, petrosal; pf, pyriform fenestra; pgf, postglenoid foramen; pgp, postglenoid process; pop, paroccipital process; pr, processus recessus; ptp, posttympanic process of the squamosal; rtp, rostral tympanic process; san, sulcus for the auricular branch of the vagus nerve (CN X); sf, stapedius fossa; sff, secondary facial foramen; smn, stylomastoid notch; Sq, squamosal; th, tympanohyal; tt, tegmen tympani

**Fig. 6:**
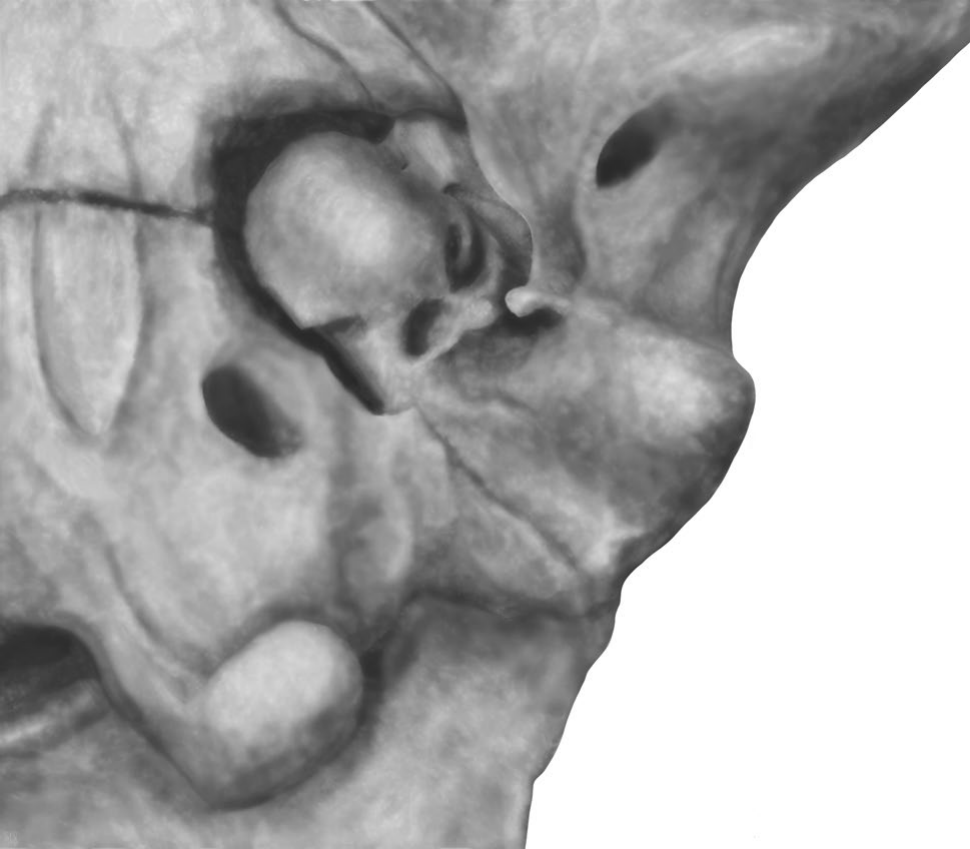
Drawing of a reconstruction of the left auditory region of *Deltatherium fundaminis* (AMNH 16610)

The left petrosal is largely intact (Figs. 3, 5 and 6). A small piece of the promontorium anterior to the external aperture of the fenestra cochleae is missing. The medial margin of the petrosal is bordered by a wide vacuity where the basisphenoid and basioccipital have been displaced; however, it is possible to tentatively infer the life position of these bones based on what is preserved given that the lateral borders of the basisphenoid and anterior part of the basioccipital are reasonably intact and preserve the natural edges of those bones on both sides. The displacement of the basisphenoid and basioccipital can be gauged from the broken bone and the position of the occipital condyle. Anteriorly, the tympanic process of the alisphenoid is displaced relative to the basisphenoid and squamosal, with its posterior edge slightly overlying the tegmen tympani of the petrosal. The crista parotica is damaged anteriorly, exposing the roof of the middle ear in ventral view.

The auditory region of *Deltatherium fundaminis,* NMMNH P–54104, preserves the petrosal bone and part of the squamosal (Fig. 4). The squamous part of the specimen includes the medial half of the glenoid fossa, the postglenoid and entoglenoid processes, and the posttympanic process of the squamosal. The petrosal is moderately well preserved; the promontorium is identifiable, and part of the caudal tympanic process is present. The pars canalicularis is not well preserved, but the overall proportions and positions of various features are evident.

### Comparative Description

In describing the auditory region of *Deltatherium* Matthew wrote:

“The auditory prominence [promontorium] of the petrosal is large, prominent, oval and rather uniformly convex, the fenestra rotunda [fenestra cochleae] situated at the top near the posterior end, the arterial tracks not discernible on the surface of the prominence [promontorium]. Externally lies the deep, long mesotympanic excavation [middle ear cavity], posteriorly the rather small and almost slit like foramen lacerum posterius [posterior lacerate foramen or jugular sulcus] and postero-externally the rounded, prominent stylomastoid foramen [notch].” (Matthew 1937, p. 72). 

We agree with Matthew’s observations and expand on them here.

In ventral view, the auditory region of *Deltatherium* is widely exposed in the distolateral corner of the basicranium (Figs. 3 and 4). The anterior margin of the petrosal is situated just anterior of the basisphenoid-basioccipital suture with the long axis of the petrosal orientated obliquely by approximately 35° (anteromedial–posterolateral) relative to a parasagittal plane on the anteroposterior axis of the cranium. Posteriorly, the wedge-shaped mastoid portion of the petrosal and forms a prominence at the distolateral corner of the braincase. The orientation and position of the petrosal in the cranium of *Deltatherium* is comparable to *Arctocyon* and the pantodonts *Pantolambda* and *Alcidedorbignya.* It differs from that observed in the tillodonts, *Esthonyx* and *Trogosus*, where the basicranial exposure of the squamosal is more extensive so as to displace the petrosal laterally to give a more oblique orientation and constrict the posterolateral exposure of the mastoid. In this regard, the tillodonts are notably different from *Deltatherium* and the other comparison taxa in having an anteroposteriorly short basicranium and posteriorly positioned temporomandibular joint.

In lateral view, the mastoid part of the petrosal of *Deltatherium* retains a small, triangular posterolateral exposure on the wall of the braincase, overhung by the nuchal crest in lateral view (Online Resource 1, Fig. S1). In ventral view, the mastoid of *Deltatherium* resembles *Pantolambda* in forming a strong eminence at the distolateral corner of the braincase; in both, it is more robust than in *Alcidedorbignya* and the tillodonts. This is notably different from *Arctocyon*, where the mastoid does not form the posterolateral margin of the braincase even though it is expanded. However, in lateral view, *Deltatherium* bears a greater resemblance to *Arctocyon*, as both possess an enlarged and posteriorly expanded nuchal crest that overhangs the occiput.

The petrosal of *Deltatherium* is large relative to the overall size of the cranium, but since the specimen is not complete (the premaxilla is damaged), it cannot be measured relative to cranial length. As preserved, and the tympanic exposure of the petrosal of *Deltatherium* accounts for ~15% of the anteroposterior length measured from the foramen magnum to the anterior border of the canine. This is similar to *Zalambdalestes* (~16%), slightly shorter than *Alcidedorbignya* (~20%), *Pantolambda* (~20%, although the cranium of AMNH 16663 is slightly telescoped), and *Trogosus* (17%), and longer than in *Esthonyx* (~8%) and *Arctocyon* (~9%). (Fig. 6) 

The petrosal bone comprises two gross divisions, the pars cochlearis anteromedioventrally and the pars canalicularis posterolaterodorsally, which together enclose the inner ear and form the roof of the middle ear. The shape and margins between the two parts of the petrosal are not explicitly discernible but can be broadly distinguished based on features of the inner ear and their corresponding topographic features on the tympanic surface of the petrosal. The tympanic exposure of the pars cochlearis is primarily composed of the promontorium and encloses the cochlea and saccule following De Beer (1929) and MacPhee (1981). In this definition, the concept of the pars cochlearis is based on the embryological development of the auditory region in extant mammals in which the saccular recess is poorly delimited from the cochlear duct during early development. However, the inclusion of the saccule in the pars cochlearis is contentious given the evolutionary development of the auditory region in fossil mammals and their relatives. In early mammal relatives, the saccule forms a discrete recess, functionally more closely related to the utricle, and separate from the cochlear recess; the pars cochlearis is a neomorphic structure with its first appearance postdating the first appearance of discrete saccular and cochlear recesses (Luo 2001; Schultz et al. 2017; Harper and Rougier 2019). We follow a topographic definition of the pars cochlearis to encompass the promontorium in our description of *Deltatherium*. The tympanic exposure of the pars canalicularis forms an ‘L’–shaped piece of bone on the lateral and posterior edges of the pars cochlearis and encloses the utricle and semicircular canals internally (De Beer 1929; MacPhee 1981). The tympanic exposure of the pars canalicularis of *Deltatherium* is relatively large, both laterally and posteriorly, compared to the tympanic exposure of the pars cochlearis. The expanded condition of the pars canalicularis in *Deltatherium* is also observed in *Pantolambda*, whereas in *Alcidedorbignya, Arctocyon, Esthonyx*, and *Trogosus* the exposure of the pars canalicularis is relatively more restricted. The widely exposed pars canalicularis condition observed in *Deltatherium* and the other Paleogene eutherians differs from that observed in *Zalambdalestes* and *Asioryctes,* where the pars canalicularis is restricted relative to the tympanic exposures of the pars cochlearis; specifically, the basicranial mastoid exposure of the pars canalicularis is smaller in these taxa*.*

The ventral exposure of the pars cochlearis of the petrosal is dominated by a promontorium, which in *Deltatherium* forms a reniform protuberance. In ventral view, the promontorium is anteroposteriorly longer than mediolaterally wide, although it is still relatively wide, with the widest mediolateral width equal to approximately 70% of the anteroposterior length. The proportions of the promontorium of *Deltatherium* are most similar to *Zalambdalestes*, *Asioryctes, Pantolambda*, *Protungulatum* and *Chriacus*, whereas in *Arctocyon, Esthonyx*, and *Trogosus*, the promontorium is more anteroposteriorly elongate. The posterolateral quarter of the promontorium of *Deltatherium* is bulbous where it encloses the parts of the cochlea with the greatest radii from the modiolar axis. As such, the posterolateral region of the promontorium projects slightly ventrally relative the ventral surface of the basicranium. The promontorium of *Deltatherium* appears somewhat bulbous relative to the ventral surface of the basicranium, more so than in *Pantolambda* but less than that observed in *Arctocyon* and the tillodonts. The long axis of the promontorium of *Deltatherium* is rotated medially by approximately ~30 degrees relative to a parasagittal plane; this is approximately the same angulation as *Arctocyon* and is slightly less than in *Zalambdalestes* and *Asioryctes* (35°), *Alcidedorbignya, Esthonyx*, and *Pantolambda* (45°), and *Trogosus* (50°). The anterior margin of the promontorium of *Deltatherium* is rounded, comparable to the condition in *Protungulatum, Chriacus, Alcidedorbignya* and *Pantolambda*; in *Arctocyon* and the tillodonts, the promontorium is more tapered to form a pyriform profile. The promontorium of *Zalambdalestes* and *Asioryctes* is also rounded but differs in that these taxa possesses a small but distinct medial shelf projecting medially around the entire anteromedial edge of the promontorium, within the same horizontal plane as the tympanic surface.

The anterior and medial borders of the promontorium of *Deltatherium* are delimited by a vacuity. A space in the chondrocranium between the otic capsule and the central stem is widespread in extant mammals during development but may not be retained in adults (Terry 1917; McDowell 1958; MacPhee 1981; Wible 2008). In *Deltatherium*, a vacuity is present in the adult. The anterorolateral part of the vacuity is formed by the pyriform fenestra between the petrosal and the sphenoid. Laterally, there appears to be very little, if any, contribution of the squamosal to the lateral margin of the pyriform fenestra, although we note that these bones have been slightly displaced. Medially, the pyriform fenestra is contiguous with the carotid opening, which in *Deltatherium* is demarcated by shallow notch in the basisphenoid rather than a discrete foramen.

The open morphology of the pyriform fenestra plus carotid notch observed in *Deltatherium* is also observed in *Pantolambda, Alcidedorbignya*, and *Trogosus*. In *Zalambdalestes,* by contrast, the carotid opening forms a discrete foramen in the basisphenoid and is separate from the pyriform fenestra, which forms an opening on the anterolateral side of the promontorium. A similar discrete carotid opening is also likely present in *Arctocyon.* The size of the pyriform fenestra in *Deltatherium* is based on a reconstruction of the specimen. However, we are confident in our inferences that a vacuity was present in life given that the sphenoid and basioccipital preserve natural edges and that they piece together well with the cranium as a whole. As such, the vacuity in *Deltatherium* is most like the condition in *Alcidedorbignya*, smaller than that observed in *Pantolambda* (with a proportionally smaller pyriform fenestra) but larger than that observed in the tillodont *Trogosus.*

In *Deltatherium,* the pyriform fenestra and carotid notch appear to be medially continuous with the basicochlear fissure (= basicapsular fenestra of MacPhee 1981). There may be a small bony stump on the anterolateral corner of the basioccipital indicating the presence of an anterior basicapsular commissure, but preservation of the specimen precludes a confident observation. A confluent pyriform fenestra, carotid notch, and basicochlear fissure is present in *Pantolambda, Alcidedorbignya*, and *Trogosus*, so a similar condition in *Deltatherium* would not be unexpected*.* Posteriorly, the basicochlear fissure in *Deltatherium* would have formed an open slit along the medial margin of the promontorium running from the carotid notch and pyriform fenestra (or anterior commissure) to the jugular foramen. In life, this may have resulted in the basicranial exposure of the inferior petrosal sinus (MacPhee 1981). An open basicochlear fissure is present in the chondrocranium between the otic capsule and parachordal plate but is often obliterated during later stages of development (De Beer 1937; MacPhee 1981). Based on AMNH 16610, an open basicochlear fissure was likely present in adult *Deltatherium* given the size and position of the basisphenoid and basioccipital in the cranium. Both the basisphenoid and basioccipital preserve natural edges, indicating that they were not in contact with the petrosal, and there is no indication of a discrete petrobasilar sulcus or canal for the inferior petrosal sinus on the dorsomedial edge of the promontorium. An open basicochlear fissure is also present in *Alcidedorbignya*, *Pantolambda*, and *Trogosus.* The preservation of the cranium of *Esthonyx* precludes observation of this feature, and the morphology in *Arctocyon* is uncertain; a reduced fissure may have been present but was likely not continuous with the pyriform fenestra. This contrasts with the condition in *Zalambdalestes*, where the petrosal contacts the basioccipital and the inferior petrosal sinus is enclosed within an endocranial sulcus.

In *Deltatherium,* the jugular foramen forms a posteriorly elongate opening that posteromedially delimits the promontorium. In life, the internal jugular vein exited the cranial cavity via this opening alongside the glossopharyngeal, vagus, and spinal accessory nerves (Cranial Nerves IX, X, and XI), indicating that this opening is equivalent to the posterior lacerate foramen (McDowell 1958; MacPhee 1981). An elongate jugular foramen is observed in all Paleogene comparison taxa where the region is sufficiently preserved, whereas in *Zalambdalestes*, the jugular foramen is demarcated by a circular opening at the posteromedial corner of the promontorium. In *Deltatherium* as well as *Arctocyon*, the size of the opening is relatively broader and slightly more open compared to the more fissured opening observed in the pantodonts and tillodonts*.* The anterior extent of the jugular foramen in *Deltatherium* is approximately level with the external aperture of the fenestra cochleae laterally, similar to the condition observed in *Arctocyon* and *Alcidedorbignya* and more anterior than the jugular foramen of *Zalambdalestes*, *Pantolambda*, and *Trogosus*. In *Deltatherium,* the posterior border of the jugular foramen extends to the level of the posterior margin of the stylomastoid notch, comparable to that observed in *Alcidedorbignya* and *Trogosus* but less posteriorly expanded than in *Arctocyon* and *Pantolambda*. There is no indication of a posterior basicapsular commissure separating the basicochlear fissure from the jugular foramen in *Deltatherium* or any of the comparison taxa, although we note the surface of the occipital bone is not well-preserved.

The surface of the promontorium of *Deltatherium* features several small outgrowths. Anterolaterally, a small epitympanic wing is present, extending laterally from the promontorium towards the pars canalicularis. The development of the epitympanic wing of the petrosal is highly variable in mammals but is defined as an outgrowth from the promontorium, separate from the tegmen tympani, anywhere along the anterolateral margin of the promontorium between the anterior margin of the promontorium and the secondary facial foramen (MacPhee 1981). In *Deltatherium*, it is anteroposteriorly restricted and does not extend to the mediolateral plane of the anterior border of the promontorium or the tegmen tympani. In *Deltatherium*, this outgrowth is very similar in size and position to that of *Pantolambda*, both of which are more laterally extensive than in *Alcidedorbignya* and *Protungulatum.* The plane of the epitympanic wing of *Deltatherium* is also most comparable to *Pantolambda,* whereas it is steeper and directed more laterodorsally in *Alcidedorbignya, Arctocyon*, and *Trogosus*. The morphology of the epitympanic wing in *Deltatherium* and the other Paleogene eutherians differs from that observed in *Zalambdalestes*, where it forms a small anteriorly projecting flange which tapers to a point anteriorly.

In the posteromedial region of the promontorium of *Deltatherium*, there is a small rostral tympanic process, a periosteal outgrowth of the pars cochlearis (MacPhee 1981; Wible and Shelley 2020). The rostral tympanic process forms a flange-like growth along the posteromedial flank of the promontorium from the anterior border of the external aperture of the canaliculus cochleae to the anterior border of the promontorium. It projects slightly ventrally in an oblique plane to the surface of the promontorium and approximates the medial margin of the basioccipital, separated from it by the basicochlear fissure. It is more prominent posteriorly, with the growth becoming flush with the promontorium anteriorly to form a medial shelf. The morphology of the rostral tympanic process of *Deltatherium* is similar to that of *Arctocyon* and *Pantolambda*. All three of these taxa resemble *Alcidedorbignya* except for their more robust process, though *Pantolambda* is further distinguished by having the plane of growth of the process closer to the surface of the promontorium*.* There is a slight swelling in the position of the rostral tympanic process in *Chriacus* and *Protungulatum*, but not enough to consider it a process, and a process is absent in *Zalambdalestes* and *Asioryctes,* although the Cretaceous taxa do possess a well-developed medial shelf along the entire medial edge of the promontorium.

The surface of the promontorium of *Deltatherium* is smooth and lacks any sulci; there is no indication of a sulcus for the internal carotid or a transpromontorial sulcus anterior to the fenestra vestibuli. However, there is a faint notch in the external aperture of the fenestra vestibuli for the stapedial artery. A shallow sulcus for the internal carotid has been reported for some specimens of *Alcidedorbignya* (Muizon et al. 2015) and is very weakly developed in *Pantolambda.* On the other hand, a marked transpromontorial sulcus is present in *Pantolambda* for the passage of the common carotid artery and stapedial arteries, but it is absent to only weakly developed in *Alcidedorbignya.* A transpromontorial sulcus is present in *Protungulatum*, and the sulci are well-developed in *Chriacus.* The promontory surface of *Trogosus* is smooth and lacks any well-defined sulci. In *Zalambdalestes*, a short sulcus is present on the dorsolateral border of the fenestra vestibuli for the passage of the stapedial artery, but a transpromontorial sulcus is absent in this taxon as well as in *Asioryctes.*

The anterolateral region of the promontorium of *Deltatherium* is shallowly excavated to form a fossa for the attachment of the tensor tympani muscle. The margins of the fossa are not well delimited. The extent and demarcation of the fossa for the tensor tympani is variable among mammals but is positioned anterior to the fenestra vestibuli and medial to the epitympanic recess (MacPhee 1981; Evans and De Lahunta 2013). In *Deltatherium*, the fossa is approximately equal to one eighth of the area of the whole promontorium. The position of the fossa in *Deltatherium* is comparable to all the comparison taxa observed. The area of attachment is not particularly pronounced in *Deltatherium,* most similar in form to that observed in *Alcidedorbignya* and *Arctocyon;* in *Zalambdalestes, Pantolambda*, *Arctocyon*, and *Chriacus*, the fossa appears more excavated, whereas in *Protungulatum*, it is strongly reduced/absent. In *Pantolambda, Arctocyon*, and particularly *Zalambdalestes*, the fossa is mediolaterally broad and well demarcated.

The posterior part of the ventral surface of the promontorium of *Deltatherium* features three openings and associated fossulae. The lateralmost of these is the fenestra vestibuli, situated within an anteroposteriorly elongate ovoid fossula on the posterolateral corner of the promontorium, which in life would have lodged the footplate of the stapes (not preserved). The morphology of the fossula of the fenestra vestibuli of *Deltatherium* is remarkably similar to *Pantolambda.* Both possess an anteroposteriorly elongate fossula, and the part of the promontorium just anterior to the fenestra vestibuli is shallowly excavated to give a sigmoidal profile to the lateral margin of the promontorium. In *Alcidedorbignya, Chriacus*, and *Protungulatum*, in contrast, the fossula is mediolaterally broader and the lateral margin of the promontorium is not excavated. In *Zalambdalestes*, the fenestra vestibuli is also an elongated oval opening recessed in a fossula, but in this taxon, it faces anteroventerolaterally and the fossula is positioned further medial on the promontorium. We refrain from providing a stapedial ratio until AMNH 16610 has been successfully CT scanned.

The fenestra cochleae is located posteromedial to the fenestra vestibuli in *Deltatherium,* within a large fossula. The external aperture of the cochlear fossula forms an oval opening orientated obliquely relative to the mediolateral axis of the basicranium so that the long axis is orientated posteromedially-anterolaterally. The aperture faces posteroventrally. The fenestra cochleae is not well delineated within the cochlear fossula due to matrix infill. The size of the external aperture of the cochlear fossula is relatively large compared to the size of the promontorium in *Deltatherium*, most similar to *Chriacus* and proportionally larger than the fossula observed in *Pantolambda* but smaller than in *Zalambdalestes.* The orientation of the external aperture fossula in *Deltatherium* is unusual in that the long axis of the opening is strongly oblique, whereas in all the other comparison taxa including *Zalambdalestes* (where the petrosal is preserved in the cranium), the aperture is orientated so the long axis of the opening is more mediolateral. In *Deltatherium,* the fenestra vestibuli and the fenestra cochleae (and their associated fossulae) are separated by a mediolaterally broad crista interfenestralis that is directed obliquely (anteromedial-posterolateral) in the same horizontal plane as the promontorium, making it contiguous with the caudal tympanic process. A mediolaterally broad crista interfenestralis is also present in *Pantolambda, Alcidedorbignya, Chriacus*, and *Protungulatum*, where it extends obliquely in the same horizontal plane as the promontorium. In *Zalambdalestes,* the crista interfenestralis is mediolaterally narrower and within the same horizontal plane as the promontorium but is directed more posteriorly, and it is also contiguous with the caudal tympanic process. A more robust crista interfenestralis is present in *Asioryctes* compared to *Zalambdalestes.*

Anteromedial to the external aperture of the cochlear fossula in *Deltatherium* is a third fossula: the external aperture of the canaliculus cochleae, which houses the ventral opening of the perilymphatic duct and forms a triangular depression on the medioventral aspect of the promontorium, slightly anterior to the external aperture of the cochlear fossula. The fossula opens towards the jugular sulcus but not into it and maintains a substantial ventral exposure. A large, ventrally facing aperture for the canaliculus cochleae is also present in *Alcidedorbignya, Esthonyx* and *Trogosus* but is notably absent in *Protungulatum, Zalambdalestes* and *Asioryctes.* In *Deltatherium,* the external aperture of the canaliculus cochleae is separated from the external aperture cochlear fossula by a mediolaterally broad and anteroposteriorly elongate processus recessus (De Beer 1929). The processus recessus is slightly obliquely orientated (anterolateral-posteromedial) and forms the lateral wall of the canaliculus cochleae fossula and the medial wall of the cochlear fossula. In *Deltatherium*, the processus recessus is approximately subequal in width to the crista interfenestralis, whereas in *Pantolambda* the processus recessus is broader and approximately twice as wide as the crista interfenestralis.

The pars canalicularis of *Deltatherium* surrounds the lateral and posterior edges of the pars cochlearis. Posterolateral to the epitympanic wing and recessed from the anterior border of the pars canalicularis, a ventral opening for the hiatus Fallopii is tentatively inferred for the exit of the greater petrosal nerve. In *Alcidedorbignya* and *Pantolambda,* the hiatus Fallopii is similarly recessed so as to open ventrally in a more posterior position, whereas in *Protungulatum* and *Zalambdalestes* the opening is positioned anteroventrally on the anterior border of the pars canalicularis. Posterior to the hiatus Fallopii in *Deltatherium*, an opening is observed just anterior to the fenestra vestibuli, the secondary facial foramen for the exit of the facial nerve from the cavum supracochleare. The position and size of the secondary facial foramen of *Deltatherium* is remarkably like that observed in *Pantolambda* and is smaller than the opening in *Zalambdalestes, Alcidedorbignya*, and *Protungulatum*, where the secondary facial foramen is at least half the size of the fenestra vestibuli*.* It is likely also smaller than the opening in *Chriacus*, but both petrosals are damaged in the vicinity of the opening, so this is inferred based on the surrounding anatomy.

On the anterolateral part of the tympanic surface of the petrosal is the tegmen tympani, a neomorphic element of the auditory capsule that originates in continuity with the anterolateral part of the pars canalicularis (De Beer 1937; Kuhn 1971; MacPhee 1981; Sánchez–Villagra and Forasiepi 2017). In *Deltatherium*, the tegmen tympani forms a broad, ventrally facing shelf that is positioned anterior to the crista parotica and lateral to hiatus Fallopii. It is subequal to half the mediolateral width of the promontorium and extends anteriorly to approximately the same point as the promontorium so as to contact the alisphenoid. The shape, size, and plane of the tegmen tympani of *Deltatherium* are very similar to what is observed in *Pantolambda* and *Arctocyon*, whereas in *Alcidedorbignya*, the ventral surface of the tegmen tympani is orientated lateroventrally and is less extensive anteriorly. In *Trogosus*, the tegmen tympani approaches the same dimensions as in *Deltatherium* but is noticeably more inflated. *Chriacus* also exhibits a slightly inflated tegmen tympani. In *Protungulatum* and *Zalambdalestes*, the tegmen tympani is comparatively reduced in anteroposterior and mediolateral dimensions.

Based on AMNH 16610, a foramen for the ramus superior of the stapedial artery is present in *Deltatherium*; it is located in the tegmen tympani, medial to the hiatus Fallopii, at the anterior end of an anteroposteriorly directed sulcus. It is partially overlain by the tympanic process of the alisphenoid, so it is not clear whether the opening is contained within the tegmen tympani or the petrosal-squamosal-alisphenoid suture. A foramen piercing the tegmen tympani is also present in *Arctocyon* and *Trogosus* but is in a relatively more posterolateral position than that inferred for *Deltatherium*. In *Pantolambda,* in contrast, a foramen is present in a comparable position (albeit slightly more anterolateral) to that inferred for *Deltatherium*, between the petrosal, squamosal and possibly alisphenoid. In *Alcidedorbignya,* the opening is almost entirely enclosed in the tegmen tympani, although bony contributions to the foramen vary across individuals. In *Zalambdalestes*, a foramen is present laterally in the petrosal-squamosal suture just anterior of the secondary facial foramen in a coronal plane. Our inferences regarding the position of the foramen for the ramus superior is tentative given the preservation of AMNH 16610 and will need to be confirmed with CT data.

The crista parotica is a distinctive longitudinal crest on the pars canalicularis (De Beer 1937). In *Deltatherium*, it is not well preserved in either AMNH 16610 or NMMNH P-54104, although its position can be deduced. Anteriorly, the crista parotica would have been continuous with the preotic crest, at the suture between the entoglenoid process of the squamosal and the alisphenoid (McDowell 1958). Within the middle ear, the tegmen tympani extends anterolaterally from the crista parotica. Posterior to the tegmen tympani, the crista parotica serves to demarcate the lateral margin of the facial sulcus and the medial border of the epitympanic recess. The tympanohyal, the most proximal ossification of Reichert’s cartilage after the stapes, is fused to the crista parotica at the posteromedial border of the epitympanic recess. The ventral extent of the crista parotica in *Deltatherium* between the preotic crest anteriorly and the tympanohyal posteriorly remains unknown, although given the ventral projection of the tympanohyal and the position of the tympanic process of the alisphenoid, it is unlikely that the crista parotica would have formed an extensive ledge. Posterior to the tympanohyal, the crista parotica is barely discernible and forms a weak ridge to the mastoid process on the posterolateral corner of the braincase, well separated from the caudal tympanic process. Based on the position and morphology of the tympanohyal relative to the roof of the middle ear cavity, it appears that the crista parotica of *Deltatherium* would have formed a distinct low ridge where it bordered the facial sulcus. The crista parotica of *Deltatherium* was likely less developed than the crista parotica observed in *Alcidedorbignya, Arctocyon*, and *Chriacus*, in which it forms a more ventrally prominent ridge. In this regard, the crista parotica of *Deltatherium* bears a greater resemblance to *Pantolambda* and *Trogosus,* although in *Trogosus* the suppression of the crista parotica is likely due to the inflation of the tegmen tympani anterior to it and the orientation of the petrosal within the basicranium. The crista parotica in *Deltatherium* was likely more developed than in *Zalambdalestes* and *Protungulatum*, both of which possess relatively low crests demarcating the lateral border of the of facial sulcus that are not well demarcated anteriorly.

The tympanohyal of *Deltatherium* forms a prominent, ventromedially directed, hook-like projection. The apex of the hook is slightly bulbous and is directed posteromedially to approximate the medial part of the lateral caudal tympanic process and form the floor of the stylomastoid notch. The ventral surface features a convex facet, which we infer is for the articulation of the stylohyal. The apex and articular facet are less robust in *Deltatherium* than in both the pantodonts and tillodonts, potentially implying a daintier hyoid apparatus, although the tympanohyal is not as gracile as in *Arctocyon* (in which it is less medially extended and has a less expanded apex). In *Deltatherium*, *Zalambdalestes, Alcidedorbignya*, and *Pantolambda*, the apex of the tympanohyal is directed posteromedially, whereas it is directed medially in *Arctocyon* and *Esthonyx* and posteromedially in *Trogosus*. The anterior flank of the tympanohyal in *Deltatherium* is smooth and forms an articular facet for the posterior crus of the ectotympanic. An indentation is evident on the posterior aspect of the base of the tympanohyal for the facial nerve and chorda tympani. Upon exiting the tympanic cavity via the stylomastoid notch, the facial nerve would have run laterally in a shallow sulcus and given rise to the chorda tympani; the chorda tympani itself would have wrapped around the tympanohyal, guided by the indentation, and run anteriorly through the Glaserian fissure (MacPhee 1981). A deeper pair of sulci are observed in this position in *Trogosus.*

Within the middle ear of *Deltatherium* is a small secondary facial foramen; it is positioned just anterior to the fenestra vestibuli and opens posteriorly into a broad facial sulcus approximately twice the size of the fenestra vestibuli in mediolateral width. The proportions of the facial sulcus in *Deltatherium* are comparable to those in *Zalambdalestes, Asioryctes, Protungulatum, Pantolambda*, and *Chriacus,* all of which have broader sulcus than that observed in *Alcidedorbignya, Arctocyon*, and *Trogosus.* Of special note in *Trogosus* is the depth of the facial sulcus, which is enhanced by laterodorsal-medioventral orientation of the petrosal in the basicranium and the inflation of the tegmen tympani and epitympanic wing relative to the size of the promontorium. The facial sulcus extends posteriorly around the posterolateral margin of the promontorium through the stylomastoid notch, lateral to the stapedius fossa. The lateral margin of the facial sulcus, between the tegmen tympani anteriorly and the tympanohyal posteriorly, is weakly defined by the crista parotica. However, the crista parotica is broken at this section and in life would have formed a more raised crest, although a low-lying crest is present in *Zalambdalestes* and *Protungulatum*

The stylomastoid notch of *Deltatherium* is large, with its greatest mediolateral width approximately equal to half the greatest mediolateral width of the promontorium. The medial wall is formed by the medial part of the lateral caudal tympanic process, the anterolateral wall is formed by the tympanohyal, and the posterior margin is weakly delimited by the lateral part of the lateral caudal tympanic process. The posteromedioventral portion of the stylomastoid notch, posterior to the fenestra vestibuli, bears a fossa for the stapedius muscle. The anterolateral portion allows for the passage of the facial nerve as it exits the tympanic cavity laterally. The margin between the stapedial fossa and passage for the facial nerve is not well delimited in *Deltatherium.* It is not clear whether the stylomastoid notch would have been enclosed by the ectotympanic to form a foramen. In *Pantolambda*, the ectotympanic is preserved and does not enclose the stylomastoid notch to form a foramen (Muizon et al. 2015). The large size of the stylomastoid notch in *Deltatherium* is notable and most comparable to *Zalambdalestes* and *Pantolambda* (note that the stylomastoid notch in *Pantolambda* is itself notched), particularly in the mediolateral dimension of the space. In all other taxa where the tympanohyal is preserved, the profile of the stylomastoid notch is more circular. Also of note is how the posterior margin of the stylomastoid notch in *Deltatherium,* which is defined by a weak ridge, is positioned far posterior to the tympanohyal and the medial part of the lateral caudal tympanic process; as a result, the stylomastoid notch opens venteroposteriorly rather than more posteriorly as in *Pantolambda* and *Alcidedorbignya.* A slightly more posteroventral opening for the stylomastoid is also observed in *Zalambdalestes* and *Arctocyon* and was likely present in *Chriacus.*

Medial to the stylomastoid notch, the posterior margin of the promontorium of *Deltatherium* is bordered by a caudal tympanic process. The caudal tympanic process is comprised of three parts following the terminology in MacPhee (1981), which was recently used by Wible and Shelley (2020). In *Deltatherium*, the medial caudal tympanic process borders the posterior margin of the external aperture of the cochlear fossula. It forms a rounded ridge that is laterally contiguous with the lateral caudal tympanic process.

The lateral caudal tympanic process is divided into medial and lateral parts based on their position relative to the stapedius fossa (MacPhee 1981; Wible and Shelley 2020). In *Deltatherium,* the medial part of the lateral caudal tympanic process forms a robust tubercle that approximates the tympanohyal laterally and extends anteriorly towards the fenestra vestibuli to contact the crista interfenestralis. The lateral part of the lateral caudal tympanic process of *Deltatherium* is not as well developed, forming a shallow ridge around the stylomastoid notch.

The caudal tympanic process of *Deltatherium* is less robust than that observed in the pantodonts *Alcidedorbignya* and *Pantolambda*, in which each of the three parts forms a prominent strut but is more prominent that the ridges observed in *Arctocyon*. The lateral part of the lateral caudal tympanic process of *Deltatherium* around the stylomastoid notch is particularly weak compared to the pantodonts*. Protungulatum* also has a less developed lateral part of the lateral caudal tympanic process, but the medial caudal tympanic process is remarkably well-developed compared to both *Deltatherium* and the pantodonts. Based on tentative observations of *Trogosus* and what is preserved of *Esthonyx*, it appears that the tillodonts have a caudal tympanic process that approximates the general configuration observed in *Deltatherium*; however, it appears relatively smaller due to the enlargement of the tympanohyal*.* In these taxa, the processes form tubercles that are more bulbous than the strut-like process in *Deltatherium*, and the lateral part of the lateral caudal tympanic process is weakly developed compared to the medial caudal tympanic process and medial part of the lateral caudal tympanic process. A caudal tympanic process is present in *Zalambdalestes* and *Asioryctes* but is weaker in these taxa than in the Palaeocene eutherians, with no section forming an enlarged ridge or strut. In *Zalambdalestes,* the medial caudal tympanic process and the lateral part of the lateral caudal tympanic process form a low but mediolaterally extensive wall along the posterior margin of the middle ear.

In *Deltatherium,* a well-marked mediolateral oblique sulcus is observed between the external aperture of the cochlear fossula and the medial caudal tympanic process plus the medial part of the lateral caudal tympanic processes, which likely conveyed the tympanic nerve (a branch of the glossopharyngeal nerve, CN IX). The tympanic nerve exited the cranial cavity via the jugular foramen and was directed anterolaterally via the sulcus traversing the fenestra vestibuli before running anterior to the otic ganglion ventral to the foramen ovale. A well-defined sulcus is also present in *Alcidedorbignya, Pantolambda*, and probably in *Trogosus*, but is absent in *Zalambdalestes*, *Asioryctes, Arctocyon, Chriacus*, and *Protungulatum.* A second small sulcus is evident at the posterior border of the medial caudal tympanic process in *Deltatherium* that may have conveyed the auricular branch of the vagus nerve (CN X) from the posterior lacerate foramen medially to the facial nerve laterally (Sisson 1938; MacPhee 1981). An equivalent sulcus is present and well developed in *Pantolambda* and *Arctocyon* but is not evident in *Zalambdalestes*, *Asioryctes, Protungulatum*, or *Chriacus.*

Laterally on the pars canalicularis of *Deltatherium*, the epitympanic recess forms a rectangular depression. It appears to be consistent in dorsoventral depth along its entire length and is situated entirely within the petrosal; the petrosal-squamosal suture forms the lateral margin of the recess, delimiting it from the external acoustic meatus. Anteriorly, the epitympanic recess opens into the Glaserian fissure. Posteriorly within the epitympanic recess, the fossa incudis, which lodges the crus breve of the incus, is not well distinguished. The shallow epitympanic recess observed in *Deltatherium* most resembles the shallow recess in *Protungulatum* and *Chriacus*, whereas in *Arctocyon, Alcidedorbignya, Pantolambda*, and *Trogosus*, the epitympanic recess is dorsoventrally deeper. In *Zalambdalestes*, the depression of the epitympanic recess into the petrosal is shallow but walled off laterally by the ventrally projecting posttympanic crest. There is no indication of an epitympanic sinus in *Deltatherium*. In *Arctocyon* and *Chriacus*, part of the petrosal dorsal to the epitympanic recess is pneumatized, but although its placement it like that of an epitympanic sinus, it is not continuous with the recess. There is large postglenoid foramen for the exit of the postglenoid vein in *Deltatherium,* located anterolateral to the epitympanic recess and posteromedial to the postglenoid process. This postglenoid foramen is most like that of *Arctocyon* and other ‘condylarths’ like *Periptychus* (Shelley et al. 2018) in terms of relative size, where it is substantially larger than the external aperture of the cochlear fossula on the promontorium; it is larger than that observed in *Alcidedorbignya*, in which the postglenoid foramen is more variable in size. In contrast, the postglenoid foramen is diminutive in *Pantolambda* and *Trogosus*.

The bony portion of the external acoustic meatus is primarily formed by the squamosal in *Deltatherium*. The ectotympanic may have contributed ventrally, but this element is not preserved for *Deltatherium*. The anterior wall of the meatus is formed by the posterior root of the zygomatic arch, including a mediolaterally robust and ventrally prominent postglenoid process. The posterior wall is formed by a small posttympanic process of the squamosal that is closely appressed to the anterior wall of the mastoid process. Together, these processes form a robust mastoid protuberance. The bony extent of the external acoustic meatus of *Deltatherium* is comparable in length to that of *Zalambdalestes, Asioryctes*, the pantodonts *Alcidedorbignya* and *Pantolambda*, and the tillodont *Esthonyx*; it is substantially longer than the meatus of *Arctocyon* but is considerably shorter than the meatus of *Trogosus.*

In *Deltatherium*, the posterior portion of the pars canalicularis is formed by the mastoid region, which is greatly expanded to form a wedge-shaped exposure on the posterolateral corner of the braincase and a prominent mastoid process. The mastoid process is separate from the paraoccipital process, which is more posteromedial and separated by a broad, shallow sulcus that extends from the stylomastoid notch to the posterolateral margin of the braincase and may have conveyed the ramus posterior of the stapedial artery as inferred for *Alcidedorbignya* (MacPhee 1981; Wible 1987; Asher 2001; Muizon et al. 2015).

The mastoid region of *Deltatherium* is most similar to that of *Pantolambda* in being wedge-shaped with a mastoid process that is massive, positioned posteromedially, and separated from the paraoccipital process by a sulcus. This general form, with a mastoid eminence and separate paraoccipital process, also occurs in *Alcidedorbignya*. However, the processes are less robust and more flange-like in *Alcidedorbignya* and are separated by a more defined sulcus. As such, the basicranial exposure of the mastoid does not exhibit the same wedge shape in *Alcidedorbignya* as that observed in *Deltatherium* and *Pantolambda.* In *Arctocyon,* the mastoid is also wedge-shaped but is considerably more inflated and bulbous compared to *Deltatherium* and *Pantolambda.* The tillodonts also present a different configuration; they resemble *Alcidedorbignya* in possessing flange-like protuberances and a more uniform (non-wedged) basicranial exposure of the mastoid, but the mastoid and paraoccipital processes lack a dividing sulcus and are joined. *Zalambdalestes* and *Asioryctes* present an alternative configuration that differs from the Paleogene eutherians; there is a single protuberance on the posterolateral corner of the braincase that formed by two anteroposteriorly compressed parts: (1) the posttympanic process of the squamosal; and (2) a process referred to as the paraoccipital process by Wible et al. (2004) but that is equivalent to the mastoid eminence as used here given the anterior position of the process on the mastoid part of the petrosal.

The areas of muscle attachment on the mastoid are not well defined in *Deltatherium*, although the ventral cap of the mastoid process is missing. Nevertheless, there is no indication of the insertion of the digastric muscles. In this regard, *Deltatherium* is likely most similar to *Pantolambda*, as taxa with more crest-like processes (*Alcidedorbignya* and the tillodonts) exhibit more marked muscle attachment areas. In *Deltatherium*, the posterolateral exposure of the pars mastoidea on the posterolateral side of the braincase is small. We were unable to identify a mastoid foramen in *Deltatherium* based on the specimens available.

## Discussion

The petrosal bone is a rich and complex source of anatomical information that can provide highly informative phylogenetic characters. Therefore, describing the auditory region for enigmatic taxa such as *Deltatherium* holds promise for resolving the higher–level phylogenetic relationships of Paleocene eutherians. Previous studies have discussed character polarity of the eutherian auditory region and hypothesised on the primitive eutherian morphotype and plesiomorphic character states for Cenozoic eutherians (e.g., MacIntyre 1972; MacPhee 1981; Cifelli 1982; Wible 1983, 1987; O’Leary 2010; O’Leary et al. 2013; Muizon et al. 2015). Our aim here is to build on these discussions with insights into the variability of basicranial anatomy in Paleocene mammals and present hypotheses on character polarity for Paleocene eutherians (summarised in Table 1).

**Table 1.** Distribution of petrosal features among a sample of Paleogene and Cretaceous eutherians considered in this study. Hypothetical primitive eutherian condition is after MacPhee (1981). *denotes uncertainty given specimen damage.

	**Primitive eutherian condition**	***Zalambdalestes*** **Late Cretaceous eutherian**	***Protungulatum*** **Incertae sedis**	***Deltatherium*** **Incertae sedis**	***Chriacus*** **Arctocyonid**	***Arctocyon*** **Arctocyonid**	***Alcidedorbignya*** **Pantodont**	***Pantolambda*** **Pantodont**	***Trogosus*** **Tillodont**
**Pyriform fenestra**	Present, narrow, positioned more anterolateral, separate from carotid opening	Present, narrow, positioned more anterolateral, separate from carotid opening	-	Present, enlarged size, greater anterior expansion, confluent with carotid opening	-	Present, narrow, anterolateral to promontorium	Present, narrow, moderate size, positioned anterolateral to promontorium	Present, greatly enlarged, positioned anterior to promontorium	Present, narrow, moderate size, positioned anterolateral to promontorium
**Carotid opening**	Discrete opening in basisphenoid	Discrete opening in basisphenoid	-	Well-demarcated notch in basisphenoid, confluent with pyriform fenestra	-	Discrete opening between sphenoid and petrosal*	Well-demarcated notch in basisphenoid, confluent with pyriform fenestra	Poorly demarcated notch in basisphenoid, confluent with pyriform fenestra	Well-demarcated notch in basisphenoid, confluent with pyriform fenestra
**Basicochlear fissure**	Open fissure, not confluent with carotid opening	Enclosed endocranially	-	Open fissure*	-	-	Open fissure, narrow	Open fissure	Open fissure
**Rostral tympanic process**	Absent	Absent	Absent, very slight swelling present but not a process	Present, small, medioventrally directed flange,	Absent, very slight swelling present but not a process	Present, small, medioventrally directed flange	Present, very small flange	Present, small flange	Present, small flange
**Transpromontorial sulcus**	-	Absent	Present	Absent	Present	-	Variably present	Present	-
**Sulcus for internal carotid artery**	-	Present in petrosal-basioccipital suture	Present on lateral side promontorium	Absent	Present on lateral side of promontorium, well demarcated	-	Variably present on lateral side of promontorium	Present on lateral side of promontorium, shallow	Present
**Groove for stapedial artery on the rim of the fossula of the fenestra vestibuli**	-	Present	Present	Present	Present	Present	Present	Present	-
**Fossa for tensor tympani**	Present	Present, enlarged	Not well demarcated	Present, weak	Present, well demarcated*	Present	Present, weak	Present, well demarcated, large	Present, mediolaterally narrow*
**Canaliculus cochleae ventral exposure**	Absent	Absent	Absent	Present	-	Present	Present (visible but more medioventral)	Present (visible but more medioventral)	Present
**Medial caudal tympanic process**	Present, well developed	Present, weak	Present, well developed	Present, ridge	Present, ridge	Present, ridge	Present, well-developed strut	Present, well-developed strut	Present, bulbous
**Medial part of lateral caudal tympanic process**	Present, weak	Present, weak	Present, weak	Present, moderate tubercle	Present, weak	Present, weak	Present, well-developed strut	Present, well-developed strut	Present, bulbous
**Lateral part of the lateral caudal tympanic process**	Present, weak	Present, weak	Present, weak	Present, weak ridge	Present, weak	Present, weak	Present, well-developed ridge	Present, well-developed ridge	Present, low ridge
**Position hiatus Fallopii opening**	Anteroventral	Anteroventral	Anteroventral	Ventral, recessed from anterior margin	-	Ventral margin	Ventral, recessed from anterior margin	Ventral, recessed from anterior margin	-
**Tegmen tympani**	Present, small, uninflated	Present, small, uninflated	Present, small*, uninflated	Present, broad, uninflated	Present, broad and inflated	Present, narrow but anteroposteriorly long, uninflated.	Present, moderate size expansion, uninflated	Present, large, uninflated	Present, large, inflated
**Foramen for ramus superior of the stapedial artery**	Present, positioned laterally in petrosal-squamosal-alisphenoid suture	Present, positioned laterally in petrosal-squamosal suture	-	Present, positioned anteriorly, incises tegmen tympani. Squamosal-sphenoid contributions unclear	-	Present, positioned laterally, incises tegmen tympani, possible squamosal contribution to opening	Present, positioned laterally, variably in tegmen tympani of petrosal or petrosal-squamosal suture	Present, positioned anterolaterally, incises tegmen tympani, with squamosal and alisphenoid contributions to opening	Present, within tegmen tympani
**Tympanohyal**	Present, small, does not approximate caudal tympanic process	Present, small, does not approximate caudal tympanic process	-	Present, moderate size, approximates caudal tympanic process	Present*	Present, small, does not approximate caudal tympanic process	Present, moderate size, approximates caudal tympanic process	Present, moderate size, approximates caudal tympanic process	Present, large, approximates caudal tympanic process
**Mastoid region, basicranial exposure**	Present, small	Present, small	Present, enlarged	Present, enlarged	Present, greatly enlarged	Present, enlarged	Present, enlarged	Present, enlarged	Present, enlarged
**Mastoid process**	-	Present, small	Present, moderately enlarged	Present, enlarged	Present, greatly enlarged	Present, greatly enlarged	Present, flange-like process	Present, enlarged	Present, enlarged and mediolaterally compressed
**Paraoccipital process**	-	Small/absent	-	Present, tubercle separate from mastoid process	Present, greatly enlarged	Present, greatly enlarged and separate from mastoid process	Present, flange-like process, separate from mastoid process	Present, enlarged and separate from the mastoid process	Present, enlarged, mediolaterally compressed and merged with mastoid

Comparisons between *Deltatherium* and other Paleogene taxa show that the auditory region of *Deltatherium* appears to preserve many plesiomorphic character states, sharing many features with the hypothetical ancestral eutherian condition as reconstructed by MacPhee (1981) (Table 1). However, these comparisons also highlight some features that have a limited distribution across Placentalia and may be of phylogenetic significance, such as an enlarged pyriform fenestra that is confluent with the carotid opening. A space in the chondrocranium between the otic capsule and the central stem is widespread in extant mammals during development but may not be retained in adults (Terry 1917; McDowell 1958; MacPhee 1981; Wible 2008). However, a space is widespread in adult specimens of Paleocene mammal species (Matthew 1937; Cifelli 1982; Muizon et al. 2015; Shelley et al. 2018) but absent in Cretaceous eutherians, which implies that it may have evolved subsequently in various eutherian (placental) lineages rather than represent the plesiomorphic eutherian condition. There is variation among Paleocene eutherians in the relative size of the pyriform fenestra and carotid opening and the contributions of the surrounding bones that form the opening that may be phylogenetically significant.

A large opening for the external aperture of the canaliculus cochleae for the perilymphatic duct is present on the ventral surface of the promontorium in many Paleocene species (see Matthew 1937; Cifelli 1982; Muizon et al. 2015) but is absent in *Zalambdalestes, Protungulatum*, and *Chriacus.* In *Protungulatum* and *Chriacus*, the canaliculus cochleae is present and recessed on the medial edge of the promontorium in a much smaller fossula. Therefore, the enlarged ventral aperture observed in *Deltatherium* is likely a derived condition relative to these early-diverging eutherians but plesiomorphic relative to Paleocene eutherians (placentals). Paleocene eutherians that possess a ventral opening exhibit variation in the relative size and shape of the aperture and fossula for the canaliculus cochleae that warrants further investigation and characterization in phylogenetic datasets.

The posteromedial part of the promontorium of *Deltatherium* features a small but robust flange-like rostral tympanic process that is also present in several other Paleocene species mentioned here (Table 1) and elsewhere (e.g., Matthew 1937; Cifelli 1982). A well-developed rostral tympanic process on the posteromedial part of the promontorium is a derived feature for Metatheria. The presence, size, and development of the rostral tympanic process in eutherians is variable and a rich source of characters for phylogenetic analyses. It is absent in Late Cretaceous eutherians, and only a small swelling is present in the appropriate position in *Protungulatum* (not enough to call a process). In Paleocene eutherians that possess a rostral tympanic process, there is variability in the plane of the growth of the process and the degree of ventral projection along its anteroposterior length.

The anterolateral part of the promontorium of *Deltatherium* features a small epitympanic wing. A similarly positioned epitympanic wing is present in the other Paleocene eutherians examined here, all of which have a more laterally expanded epitympanic wing compared to the condition in *Zalambdalestes*, where the outgrowth is directed more anteriorly*.* The epitympanic wing is hugely variable in extant placentals in its extent, relationship to the rostral tympanic process and tegmen tympani, and plane of growth; therefore, capturing the variations in the size, position, and plane of the epitympanic wing in character datasets for Paleocene species (even though it relatively small) may prove fruitful. Similarly, characterizing the variation in the tegmen tympani would likely be useful given that the size and development of the tegmen tympani is an important character for distinguishing artiodactyls and perissodactyls (O’Leary 2010). *Deltatherium* possesses a mediolaterally broad tegmen tympani that extends anteriorly to approximately the same mediolateral plane as the promontorium. There is a fair degree of variability in the tegmen tympani in the Paleocene taxa considered here (and the position of the foramen for the ramus superior of the stapedial artery), all of which feature a relatively expanded tegmen tympani compared to *Zalambdalestes* and *Protungulatum.*

Also of relevance here is the development of the fossa for the tensor tympani. MacPhee (1981) hypothesised that a tensor tympani muscle is present in the primitive eutherian morphotype and subsequently reduced or lost. The morphology of *Zalambdalestes* supports MacPhee’s hypothesis; however, the reduction of the fossa in taxa such as *Protungulatum* versus retention in others such as *Pantolambda* implies a greater degree of variability than previously expected even among relatively closely related taxa (e.g., the small fossa *Alcidedorbignya* versus the relatively large fossa in *Pantolambda*).

The caudal tympanic process is also a potentially rich source of phylogenetic information. We recognize that there may be a degree of intraspecific variation (even bilaterally within the same individual) as observed in *Alcidedorbignya* (Muizon et al. 2015)*.* However, there are clear structural differences in the parts of the caudal tympanic process, from the subtle ridges observed in *Zalambdalestes* to more pronounced ridges in *Deltatherium* to strut-like growths in the pantodonts and inflated tubercles in the tillodonts. Additionally, the relative development of the three parts of the caudal tympanic process shows marked variation, with different parts being more or less developed compared to the others. 

The auditory region of *Deltatherium* conforms to the highly plesiomorphic condition described for other Paleocene eutherians in many regards (Matthew 1937; Cifelli 1982; O’Leary 2010; Muizon et al. 2015; Shelley et al. 2018), but detailed comparative description of the tympanic surface of the petrosal shows that there are subtle similarities and differences that have the potential to help inform the phylogenetic position of *Deltatherium* and other Paleocene mammals. 

## Supplementary Information

Below is the link to the electronic supplementary material.

Supplementary file1 (PDF 807 KB)

## Data Availability

All data generated or analysed during this study are included in this published article and its supplementary information files.
