# Supplementary material for: Petrosal Anatomy of the Paleocene Eutherian Mammal Deltatherium fundaminis (Cope, 1881)
Source: J Mamm Evol. 2021 Aug 31;28(4):1161–80. doi: 10.1007/s10914-021-09568-3 (PMC8406390; doi:10.1007/s10914-021-09568-3)
Supplement: Supplementary file 1 — Supplementary file1 (PDF 807 KB) [file 10914_2021_9568_MOESM1_ESM.pdf]

Online Resource 1 for:

**Petrosal anatomy of the Paleocene eutherian mammal *Deltatherium fundaminis* (Cope, 1881)**

*Journal of Mammalian Evolution*

Sarah L. Shelley[a,b1], Ornella C. Bertrand[a] Stephen L. Brusatte[a,c], Thomas E. Williamson[c]

[a]University of Edinburgh, School of GeoSciences, Edinburgh, United Kingdom.
[b]Carnegie Museum of Natural History, Pittsburgh, Pennsylvania, United States of America
[c]New Mexico Museum of Natural History and Science, Albuquerque, New Mexico, United States of America.
[1]To whom correspondence should be addressed. Email: sarah.shelley@ed.ac.uk (SLS)

This PDF file includes:
Supplementary figures S1 to S7

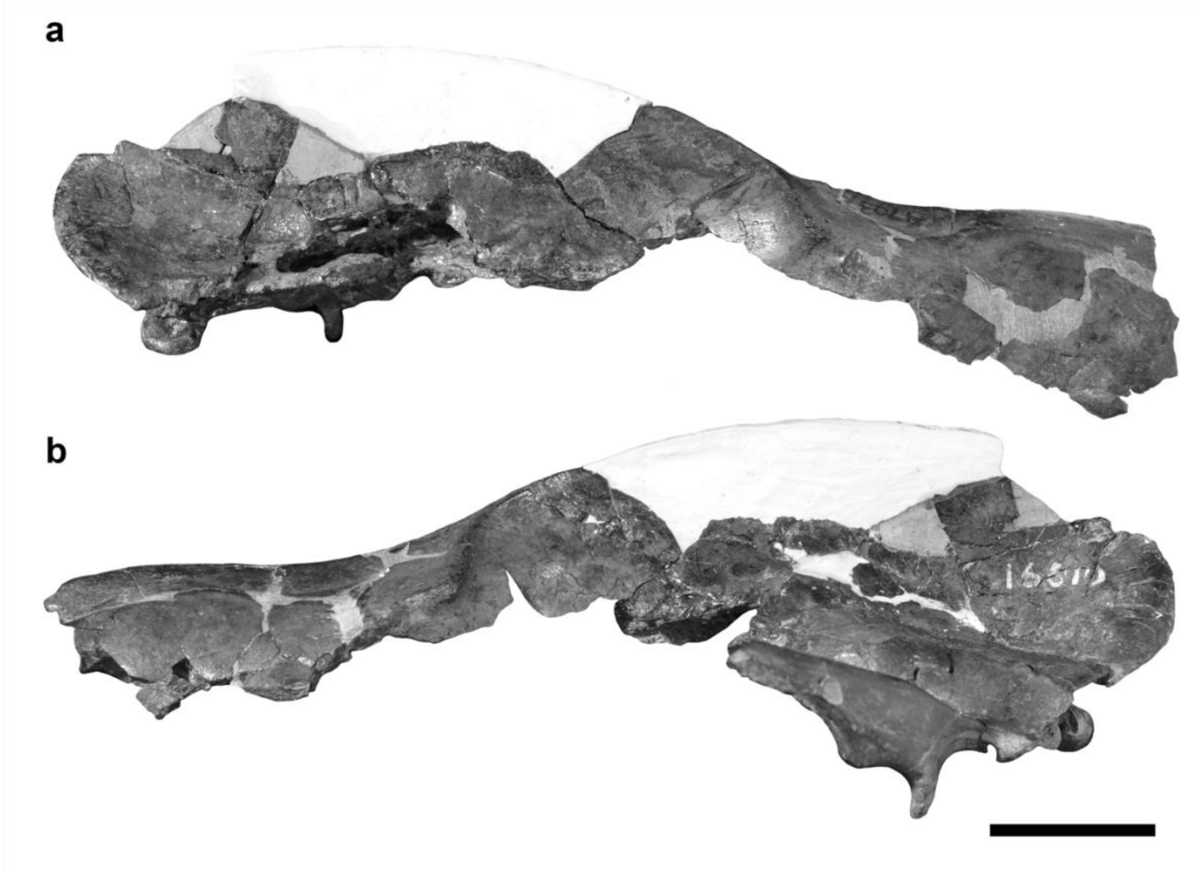


**Fig. S1** Photographs of the cranium of *Deltatherium fundaminis* (AMNH 16610) in a, right lateral view; b, left lateral view. Scale bar = 20 mm

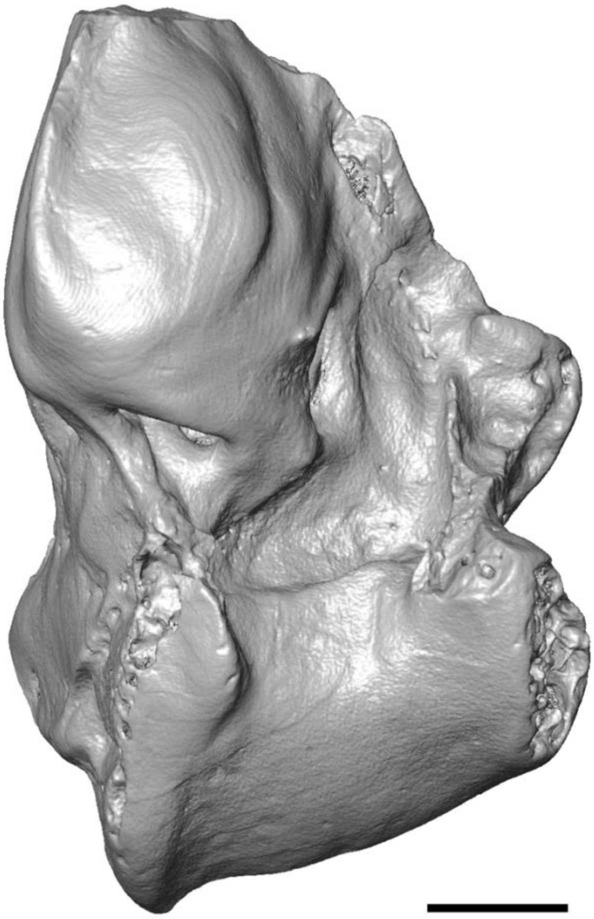

**Fig. S2** CT rendering of AMNH 118359, a left petrosal referred to *Protungulatum* sp. in ventrolateral view. Scale bar = 1 mm

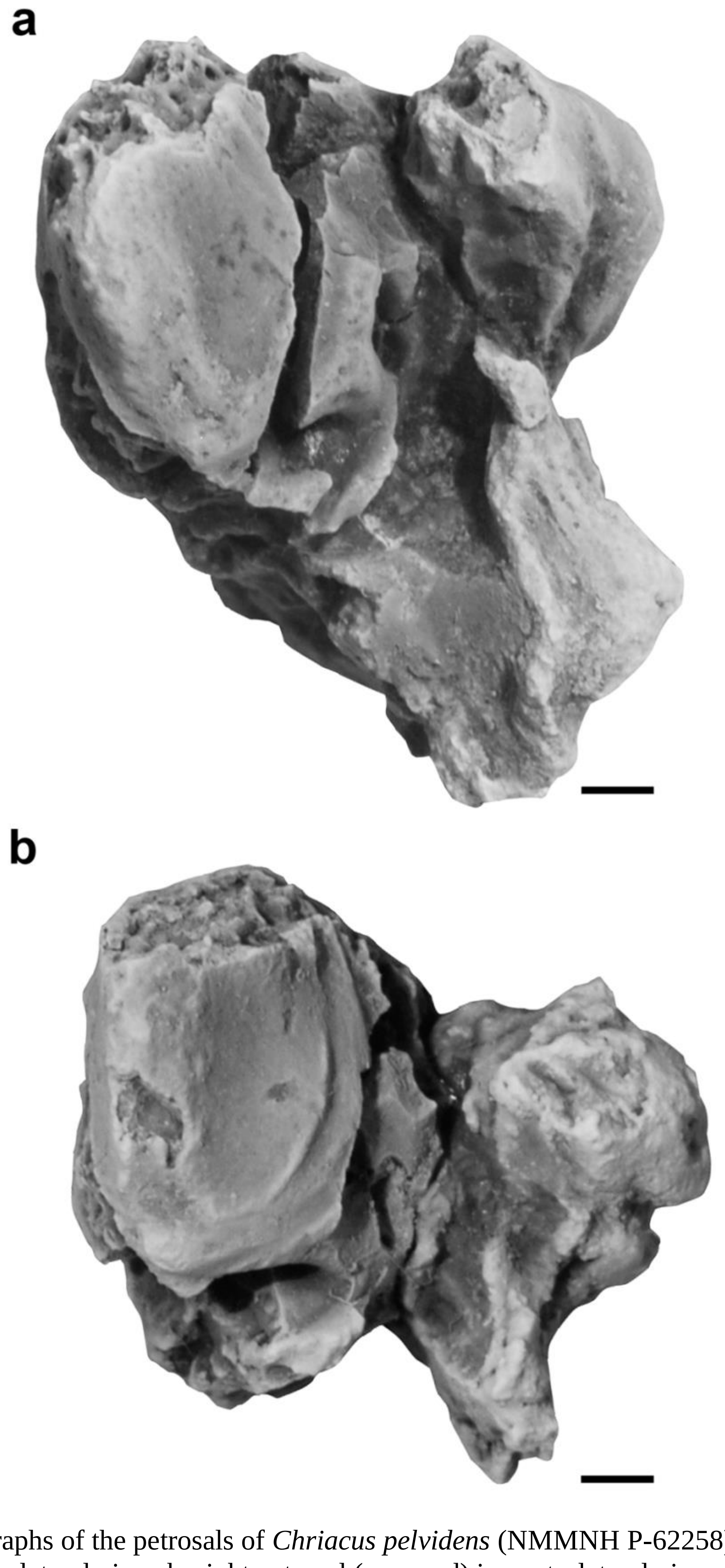

**Fig. S3** Photographs of the petrosals of *Chriacus pelvidens* (NMMNH P-62258) a, left petrosal in ventrolateral view; b, right petrosal (reversed) in ventrolateral view. Scale bars = 1 mm

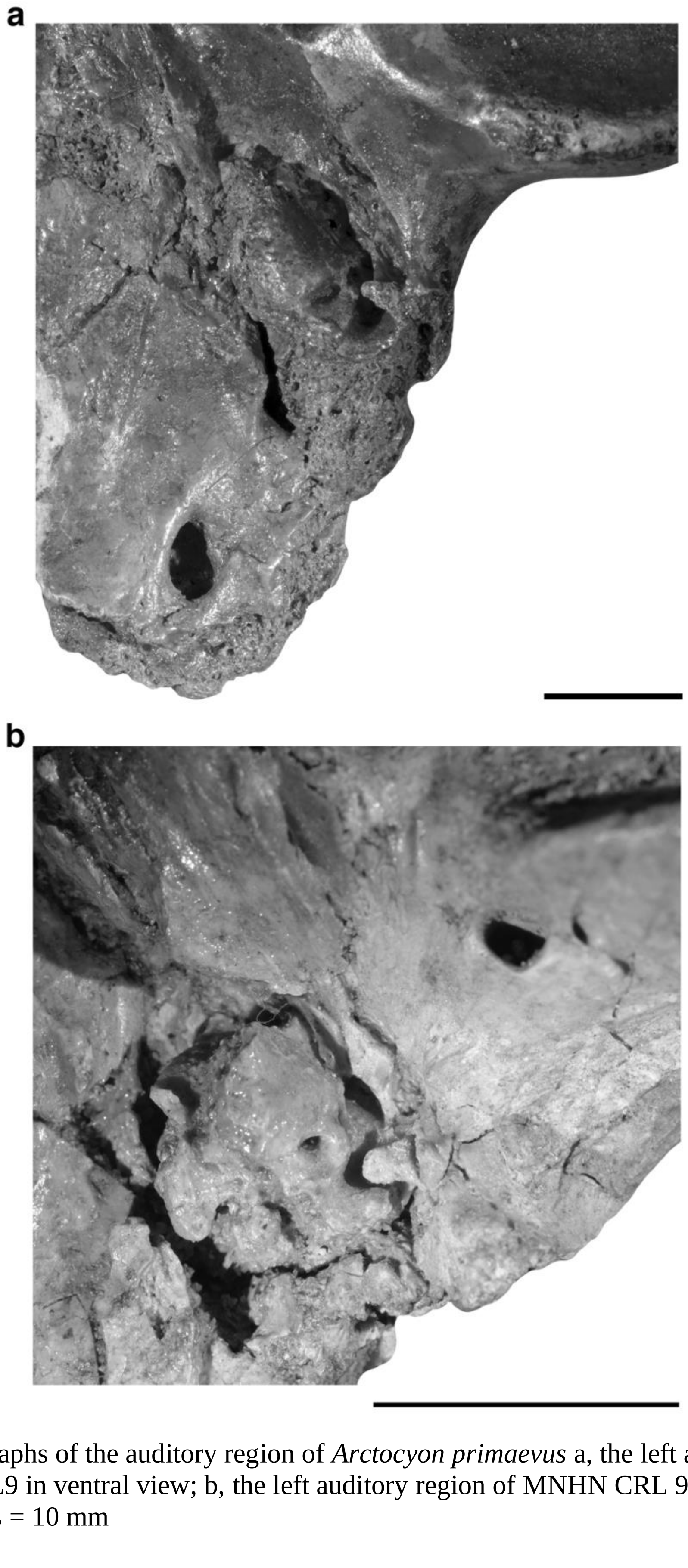


**Fig. S4** Photographs of the auditory region of *Arctocyon primaevus* a, the left auditory region of MNHN BR L9 in ventral view; b, the left auditory region of MNHN CRL 957 in ventral view. Scale bars = 10 mm

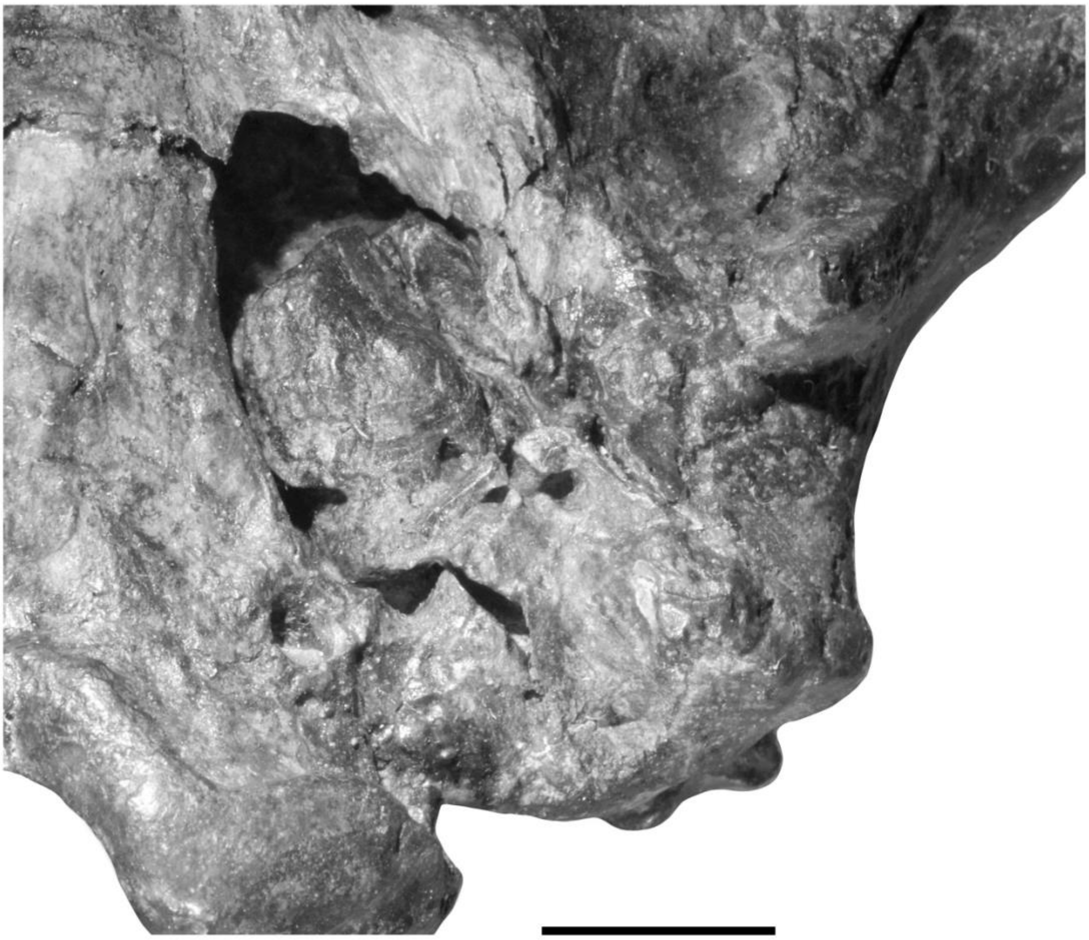

**Fig. S5** Photograph of the left auditory region of *Pantolambda bathmodon* (AMNH 16663) in ventral view. Scale bar = 10 mm

**a**

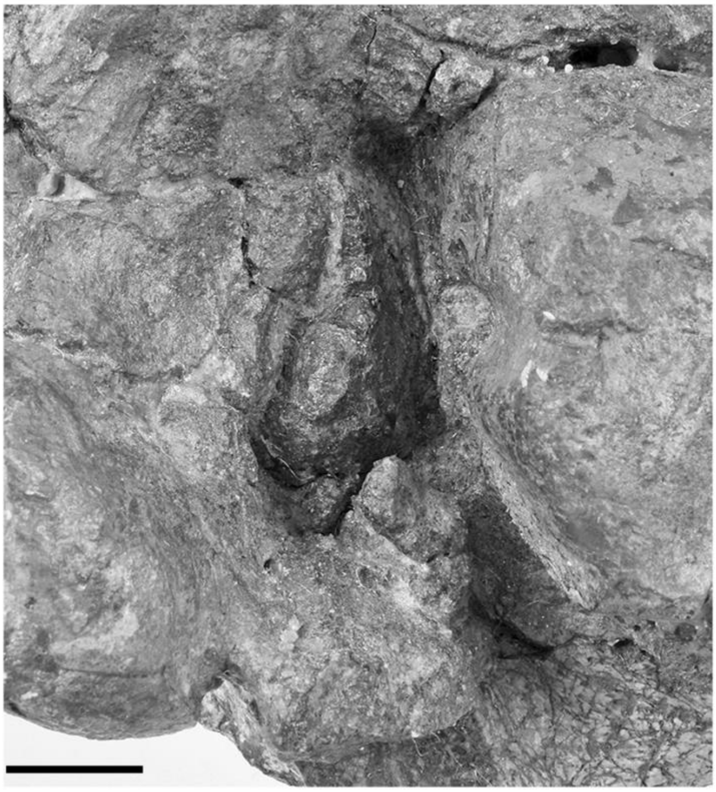

**b**

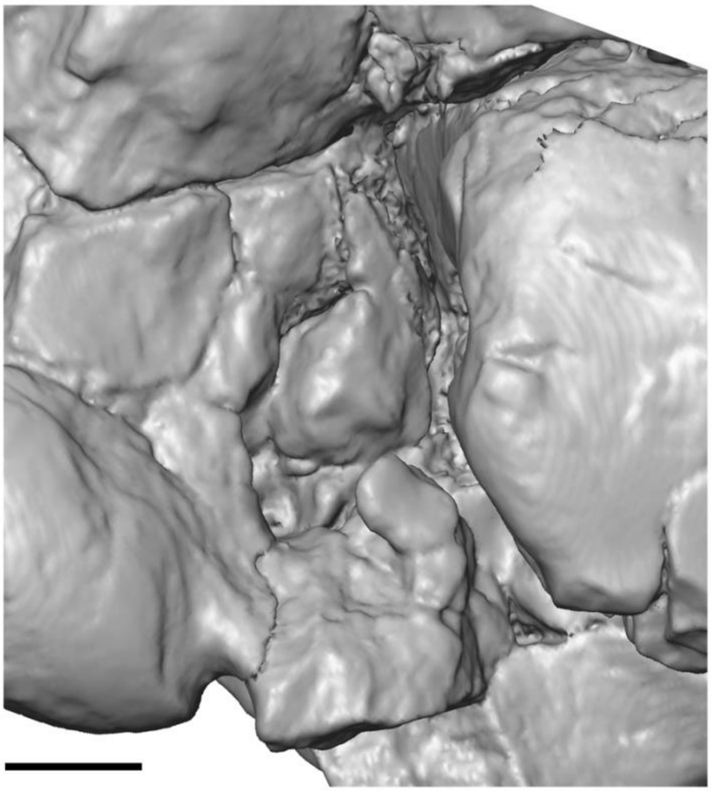

**Fig. S6** The auditory region of *Trogosus hillsi* (USNM 17157) a, photograph of the left auditory region in ventral view; b, CT rendering of the left auditory region in ventral view. Scale bars = 10 mm
